# Localization and connections of the tail of caudate and caudal putamen in mouse brain

**DOI:** 10.3389/fncir.2025.1611199

**Published:** 2025-08-04

**Authors:** Run-Zhe Ma, Sheng-Qiang Chen, Ge Zhu, Hui-Ru Cai, Jin-Yuan Zhang, Yi-Min Peng, Dian Lian, Song-Lin Ding

**Affiliations:** ^1^Key Laboratory of Neuroscience, School of Basic Medical Science, Guangzhou Medical University, Guangzhou, China; ^2^Department of Psychology, School of Health Management, Guangzhou Medical University, Guangzhou, China; ^3^Allen Institute for Brain Science, Seattle, WA, United States

**Keywords:** connectivity, caudal striatum, substantia nigra, globus pallidus, association cortex, dopamine receptor, neurotensin, *prodynorphin*

## Abstract

The neural circuits of the striatum (caudate and putamen) constitute a crucial component of the extrapyramidal motor system, and dysfunction in these circuits is correlated with significant neurological disorders including Parkinson’s disease and Huntington’s disease. Many previous studies in rodents revealed the neural connections of the rostral and intermediate parts of the striatum, but relatively fewer studies focused on the caudal striatum, which likely contains both the tail of caudate (CaT) and caudal putamen (PuC). In this study, we investigate the gene markers for the CaT and PuC and brain-wide afferent and efferent projections of the caudal striatum in mice using both anterograde and retrograde neural tracing methods. Some genes such as *prodynorphin*, *otoferlin*, and *Wolfram syndrome 1 homolog* are strongly expressed in CaT and PuC while some others such as neurotensin are almost exclusively expressed in CaT. The major afferent projections of the CaT originate from the substantia nigra (SN), ventral tegmental area, basolateral amygdala, parafascicular nucleus, and visual, somatosensory, auditory and parietal association cortices. The PuC receives its main inputs from the posterior intralaminar nucleus, ventroposterior medial nucleus (VPM), medial geniculate nucleus, and entorhinal, motor and auditory cortices. Both CaT and PuC neurons (including dopamine receptor 1 expressing ones) project in a rough topographical manner to the external and internal divisions of globus pallidus (GP) and SN. However, dopamine receptor 2 expressing neurons in nearly all striatal regions (including CaT and PuC) exclusively target the external GP. In conclusion, the present study has identified the mouse equivalent of the primate CaT and revealed detailed brain-wide connections of the CaT and PuC in rodent. These findings would offer new insights into the functional correlation and disease-related neural circuits related to the caudal striatum.

## 1 Introduction

The basal ganglia is a set of nuclei located in the forebrain base and mainly includes the striatum (neostriatum) and globus pallidus (paleostriatum). The striatum controls a series of complex functions, works in coordination with the cerebral cortex and thalamus, and is involved in many different physiological processes, especially the movement and the adjustment of learning (Fazl and Fleisher, 2018). The striatum receives extensive projections from the limbic, association and sensorimotor cortices and sends connections to the external (lateral) and internal (medial) globus pallidus (GPe and GPi, respectively), subthalamic nucleus (STh) and substantia nigra (SN) (Parent and Hazrati, 1993; Haber, 2003; Utter and Basso, 2008). There are two main excitatory pathways to the striatum, one from the cerebral cortex and the other from the thalamus, particularly the parafascicular-centromedian nuclear complex (PF-CM or PF in rodent literature). The sensorimotor pathway from the somatic/body representation targets the dorsolateral striatum (DLS) in a rough somatotopic arrangement with trunk, lower limb, upper limb and mouth regions located dorsoventrally in the DLS (Carelli and West, 1991; Hintiryan et al., 2016; Hunnicutt et al., 2016; Foster et al., 2021). The PF-striatal pathway is also topographically organized with lateral PF projects to DLS, central PF to the dorsomedial striatum (DMS) and medial PF to the medial striatum (Mandelbaum et al., 2019; Foster et al., 2021). The striatum imposes this rough topography to its target structures such as the GPe and SN (Foster et al., 2021). The striatum is often divided into the ventral and dorsal striatum (VS and DS, respectively). The VS includes nucleus accumbens (NAC) and olfactory tubercle (OT) while the DS consists mainly of the caudate (Ca) and putamen (Pu). The Pu, sometimes also referred to as the DLS, is situated in the lateral aspect of the lenticular nucleus, whereas the Ca is alternatively known as the DMS (Balleine and O’Doherty, 2010; Chen et al., 2011). The VS, DLS (Pu) and DMS (Ca) have some overlap and some distinct functions, with the VS (mainly NAC) related to rewards and motivation, the Ca related to cognition and working memory, and the Pu mainly related to motor control (Haber, 2016).

The Pu, rather than the Ca, primarily participates in regulating motor behavior of the body (Alexander et al., 1990; Haber, 2016). Damage or inactivation of the Pu region will disrupt normal body movements (Denny-Brown and Yanagisawa, 1976; Kato and Kimura, 1992; Kendall et al., 1998). Motor circuits that link the Pu to the premotor and sensorimotor cortex play a crucial role in sustaining habitual behaviors (de Wit et al., 2012; Seger, 2018), while excessive activation of Pu neurons typically leads to involuntary movements (Alexander and DeLong, 1985). Numerous studies have demonstrated that Pu dysfunction is linked to a variety of diseases, including dystonic syndrome (Todd and Perlmutter, 1998), Parkinson disease (Shen et al., 2020), attention deficit and hyperactivity disorder (Max et al., 2002), and Huntington’s disease (Coppen et al., 2018; Lee et al., 2023). However, Ca is reported to have differential functions and is more related to motor planning and flexible responding (Vandaele et al., 2021; Gould et al., 2024). Previous studies have suggested that the connections from the cerebral cortex to the Ca and Pu form parallel loops known as associative circuit and motor circuit, respectively (Middleton and Strick, 2000; Yin and Knowlton, 2006; Milad and Rauch, 2012; Seger, 2018). Hence, it can be hypothesized that neural circuits originating from the Pu and Ca may function differently (Alexander et al., 1986). It is important to note that the Ca and Pu in mouse and rat brains are not clearly separated from each other by the discrete internal capsule (ic) and thus together are termed caudoputamen (CP) or DS with the region corresponding to primate Ca located in the mediodorsal part of the CP (thus termed CPmd, or CPdm or DMS; see Mannella et al., 2013; Hintiryan et al., 2016). The CP region corresponding to primate Pu is large and located lateroventral to the CPmd (thus termed CPlv or lateral striatum; see Coffey et al., 2016). This large CPlv region is often referred to as DLS (e.g., Mannella et al., 2013; Rothwell et al., 2015) or further subdivided into dorsolateral, ventromedial and ventrolateral parts of the striatum (see Lipton et al., 2019). Additionally, mouse CP is also roughly subdivided into rostral, intermediate and caudal parts (Hintiryan et al., 2016).

Given the crucial and distinct roles of different CP regions, it is imperative to pinpoint different individual domains within this intricate CP to investigate their shared and unique neural circuits as well as potential functional significance. Most of previous connectional studies of rat and mouse CP have focused on the rostral and intermediate parts of the CP or specific pathways such as cortico-striatal projections (McGeorge and Faull, 1989; Hintiryan et al., 2016; Hunnicutt et al., 2016; Miyamoto et al., 2018). A few recent studies have examined the afferent connections of the caudal CP (so-called tail of striatum) in rats and mice (Pan et al., 2010; Jiang and Kim, 2018). However, these studies appear to treat the caudal CP as a single entity. It is still unknown if differential connections exist between the caudal dorsal and caudal ventral CP. In addition, the possible mouse equivalent of the tail of caudate in primates (CaT) has not yet identified and defined although it could be presumed as a part of the caudal CP. Additionally, mouse equivalent of the posterior (caudal) ventral Pu (PuPV or PuCv) in human (primate) brains (Ding et al., 2016) also likely exists in the caudal CP or caudal Pu (PuC). Therefore, the first aim of this study is to identify some gene markers for the CaT and/or PuC in primate and mouse brains. The second aim is to reveal detailed connections of the dorsal and ventral parts of the caudal CP, which likely correspond to the CaT and PuC, respectively. The third aim of this study is to identify potential differential connections of the caudal CP and more rostrally located intermediate CP.

## 2 Materials and methods

### 2.1 Animals

Forty adult C57BL/J6 mice weighing 20–25 g (9–12 weeks; Beijing Vital River Laboratory Animal Technology Co., Ltd., Beijing, China), half male and half female, were used in this study. All animals were kept in the same environment, ensuring appropriate temperature, controlled light, and free access to food and water. All surgery operations were performed under deep anesthesia to reduce the perception of pain. All experimental procedures were followed in accordance with the protocols that have been approved by the Institutional Animal Care and Use Committee of Guangzhou Medical University.

### 2.2 Surgery procedure and tracer injections

Before the start of the experiment, the operating table and surgical tools were disinfected with 75% alcohol. Then the mice were anesthetized with intraperitoneal injection of 3.0% pentobarbital sodium (1 ml/kg body weight). The mouse was secured in a stereotaxic frame with erythromycin covering its eyes. The bregma and lambda of the mouse skull were adjusted to the same horizontal plane and the skin was sterilized prior to creating a midline incision over the skull. After that, two burr holes (one on each side) were drilled on the skull over the target brain areas, according to the coordinates specified in the mouse brain atlas by Paxinos and Franklin (2001). The Following are the coordinates (in mm) of the target regions: two targets in intermediate CP (AP −0.58, ML 3.00, DV 3.65 or AP −0.70, ML 3.00, DV 3.60) and four targets in caudal CP (AP −0.94, ML 2.85, DV 3.55 or AP −1.22, ML 2.90, DV 3.70 or AP −1.58, ML 2.90, DV 3.75, or AP −1.70, ML 2.80, DV 3.25). Neuronal tracers of 0.05–0.06 μL 10% biotinylated dextran amine (BDA) or 4% Fluoro-Gold (FG) were delivered to the target area using 0.5 μL Hamilton microsyringe. Following the injection, the needle of the microsyringe was kept in place for 10 min before slowly pulled out. After the midline incision was stitched, the mouse was placed on the warm bed for a period and returned to its cage when it could move freely.

### 2.3 Brain preparation

The mouse was given deep anesthesia 7–10 days after surgery, and cardiac perfusion of 0.9% normal saline and 4% paraformaldehyde in 0.05 M phosphate buffered saline (PBS) was performed. Then the brain was extracted from the cranial cavity and immersed in 4% paraformaldehyde at 4°C overnight and in 15% and 30% sucrose solution in sequence for cyoprotection. The brain was cut into two halves, embedded in OCT, and cut into 40 μm thick coronal sections using a frozen microtome. All brain sections were preserved in a cryoprotectant solution in a −20°C refrigerator before use.

### 2.4 BDA tracing

The histochemistry method for BDA tracing has been described in our previous study (Chen et al., 2021; Xiang et al., 2023). First, the sections were rinsed using 0.05 M PBS (at least five times, 5 min each). And then the sections were incubated in 0.3% Triton X-100 in 0.05 M PBS for 1.5 h and in Streptavidin-Biotin Complex solution (SABC kit, Boster Biological Technology) for 3 h at room temperature. Following rinses in 0.05 M PBS, the sections were visualized with 0.05% 3, 3-diaminobenzidine (DAB) in 0.05 M PBS. Finally, the sections were mounted on chrome alum and gelatin coated glass slides, dehydrated in gradient ethanol and xylene, and coverslipped.

### 2.5 FG tracing

Selected sequential sections for FG tracing were rinsed with 0.01 M PBS and mounted on chrome alum and gelatin coated glass slides. Then the sections were examined and photographed under an upright fluorescence microscope (Leica DM6B Thunder or Axio Observer7). The brain sections were kept moist throughout these processes.

### 2.6 Semi-quantification of FG-labeled neurons

To score the relative density of labeled neurons following FG injections into the intermediate CP and rostral and caudal levels of the caudal Pu, the density of FG labeled cells in different brain regions were evaluated based on average density from 3 to 5 cases in each injection group (Supplementary Table 1). The three groups include those with FG injections into the ventral intermediate Pu (B = −0.58 or −0.70 mm), rostral level of the ventral caudal CP (B = −1.22 mm) and caudal level of the caudal CP (B = −1.58 or −0.70 mm). In addition, the last group includes dorsal (CaT∼) and whole caudal CP (CaT∼ + PuC) injection cases to explore possible differences between the dorsal and ventral caudal CP (Supplementary Table 1).

### 2.7 Image acquisition and processing

The sections with FG-labeling were photographed using fluorescence microscopy (Leica DM6B Thunder or Axio Observer7). And the sections with BDA-labeling were scanned using slide scanner (Aperio CS2, Leica). Semi-quantification of FG labeled neurons were performed using ImageJ. All selected images underwent processing in Adobe Photoshop, which included cropping, adjusting brightness and contrast, synthesizing images, and annotating structures. Additional datasets for the C57BL/J6 mice were derived from the publicly accessible Allen Institute portal^1^ and processed in a similar way.

## 3 Results

### 3.1 General subdivisions of the primate striatum and nomenclature for striatum

**FIGURE 1:**
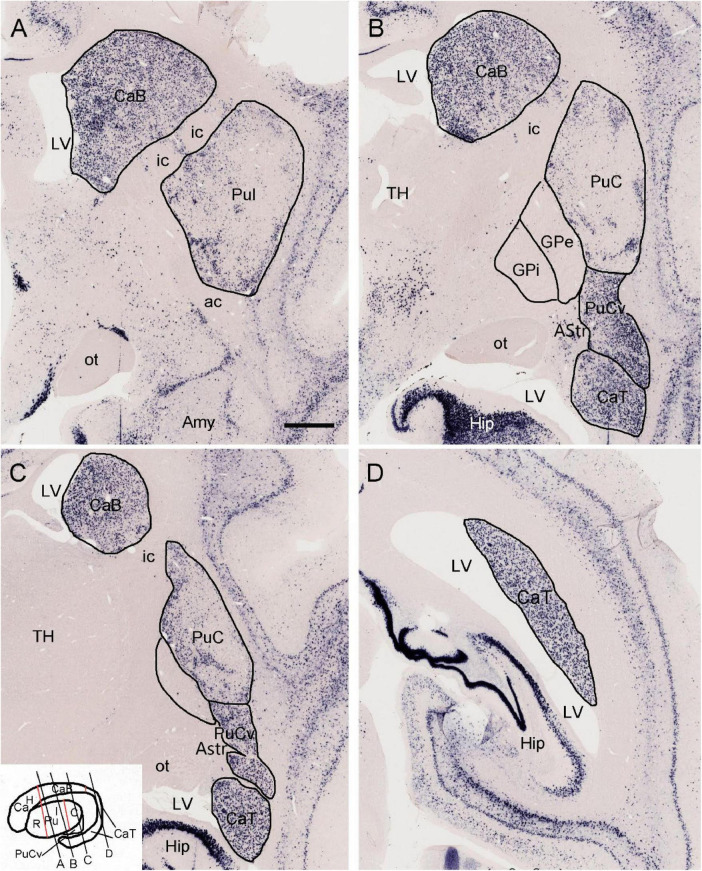
Expression of prodynorphin (*Pdyn*) in the striatum of a 1 year-old marmoset. **(A)** *Pdyn* is strongly and overall, homogenously expressed in the caudate body (CaB). In contrast, *Pdyn* is overall weakly expressed in the intermediate putamen (PuI). **(B–D)** Strong *Pdyn* expression extends caudally along the CaB and CaT (caudate tail). It is noted that while typical putamen [PuI and caudal putamen (PuC)] displays overall weak *Pdyn* expression, the ventral part of the PuC (PuCv) shows strong *Pdyn* expression **(B,C)**. Additionally, the amygdalostriatal transition area (AStr), which is located medial to the PuCv, displays weak or faint *Pdyn* expression **(B,C)**. The inset in panel **(C)** shows the major subdivisions of the primate striatum. The red lines indicate the borders between CaH (caudate head) and CaB, and between CaB and CaT, as well as the borders between rostral putamen (PuR) and PuI, and between PuI and PuC. The black oblique lines **(A–D)** indicate the approximate locations of the sections in panels **(A–D)**, respectively. For abbreviations see the list (for all figures). Bar: 730 μm in panel **(A)** for panels **(A–D)**.

**FIGURE 2:**
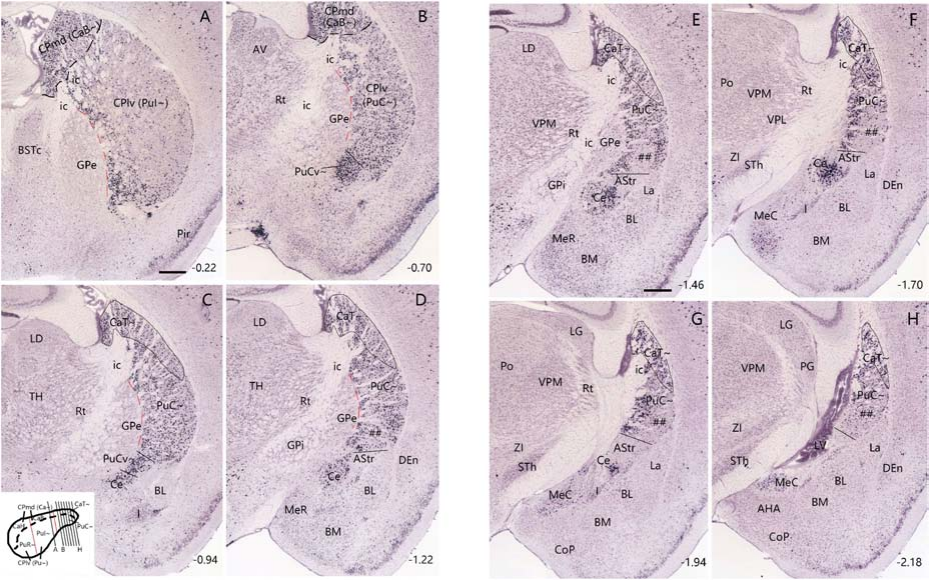
Expression pattern of *Pdyn* in the mouse striatum (case ID: 71717084). The red dashed lines indicate the borders between the Pu∼ (PuI∼ and PuC∼) and GPe. **(A)** *Pdyn* expression in the intermediate CP. *Pdyn* is densely and homogenously expressed in the mediodorsal part of the CP (CPmd), which is roughly the putative caudate (Ca∼, specifically the CaB∼). In the lateroventral part of the CP (CPlv), which is roughly the putative putamen (Pu∼, specifically the PuI∼), overall weak *Pdyn* expression is seen. **(B)** *Pdyn* expression in a section located in the junction between the intermediate CP **(A)** and caudal CP **(C)**. At this level, the weak *Pdyn*-expression zone shrinks, while the strong *Pdyn-*expression region expands. Most ventrally, a distinct strong expression region appears, probably corresponding to the PuCv described in human (Ding et al., 2016) and marmoset (see Figures 1B, C) brains. **(C–H)** *Pdyn* expression in the caudal striatum in sequential rostral-caudal coronal sections. Both dorsal (CaT∼) and ventral (PuC∼) striatal regions exhibit overall strong *Pdyn* expression, but in the ventral part of the PuC∼, a strong-weak-strong pattern of *Pdyn* expression exists mediolaterally **(D,E)**. The lateral strong expression zone disappears caudally **(F,G)**. The weak expression zone is indicated in panels **(C–G)** (by #s). Additionally, a *Pdyn*-negative zone emerges among the PuC∼, Ce and the rostral BL. This zone roughly corresponds to the AStr **(C–G)**. *Pdyn* is also strongly expressed in the central amygdaloid nucleus [Ce in panels **(C–G)**]. Approximate bregma coordinates are indicated at the bottom right corner of each panel. Bar: 420 μm in panel **(A)** for panels **(A–H)**.

**FIGURE 3:**
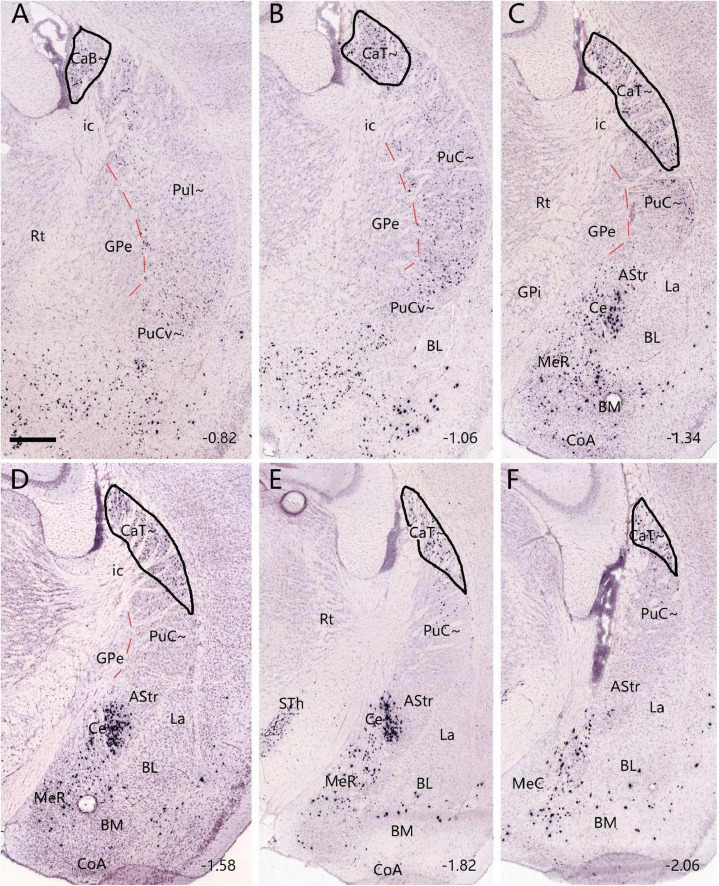
Neurotensin (*Nts*) expression in the CaB∼ and CaT∼ of the mouse (case ID: 73788032). The red dashed lines indicate the borders between the Pu∼ (PuI∼ and PuC∼) and GPe. **(A)** *Nts* expression in the CaB∼ of the intermediate CP. **(B–F)** *Nts* expression in the caudal CP. It is noted that *Nts* expression is clearly visible in the dorsolateral part of the caudal CP (CaT∼) with only faint expression in the underlying PuC∼. Approximate bregma coordinates are indicated at the bottom right corner of each panel. Bar: 420 μm in panel **(A)** for panels **(A–F)**.

To provide a context for comparative subdivisions of the striatum in the primates and rodents, we have searched the marmoset gene atlas^2^ (Kita et al., 2021) and found that the expression patterns of some genes such as *Pdyn* (prodynorphin) are very helpful in distinguishing the Ca and Pu, and their subdivisions. Specifically, for example, *Pdyn* is strongly and overall, homogeneously expressed in the VS (data not shown), entire Ca and PuCv (Figures 1A–D). In contrast, *Pdyn* expression in the typical Pu is overall weaker with some strongly labeled, scattered patches (Figures 1A–C). It is important to note that all Ca subregions along the rostral-caudal axis (i.e., head, body and tail of the Ca: CaH, CaB and CaT; see Ding et al., 2016 for human Ca subdivisions, and the inset of Figure 1C for marmoset Ca subdivisions) show strong *Pdyn* expression (Figures 1A–D). This feature can be used to identify putative Ca (Ca∼) and putative Pu (Pu∼) as well as their subdivisions in rodents (see Figures 2, 3). Similarly, primate Pu can also be subdivided into rostral (PuR), intermediate (PuI) and caudal (PuC) subdivisions along the rostral-caudal axis (see the inset of Figure 1C for marmoset Pu subdivisions).

It should be mentioned that rodents usually have fused Ca and Pu, collectively called DS or CP (Hintiryan et al., 2016). In rodent literature, DMS and DLS at rostral levels are often treated as the rodent equivalents of the primate Ca and Pu, respectively (Lee et al., 2023). Mouse DS or CP is also roughly subdivided into rostral, intermediate and caudal subdivisions (Hintiryan et al., 2016). Similarly, in the present study, we first divide the DS/CP into rostral, intermediate and caudal parts. Second, the possible mouse homolog of primate Ca is termed mediodorsal CP (CPmd) or putative Ca (Ca∼) while the mouse homolog of primate Pu is termed lateroventral CP (CPlv) or putative Pu (Pu∼). We choose to mainly use terms Ca∼ (including CaH∼, CaB∼, and CaT∼) and Pu∼ (including PuR∼, PuI∼, PuC∼, and PuCv∼) because one of our aims of this study is to identify and locate the Ca∼ and Pu∼, particularly the CaT∼, PuC∼, and PuCv∼ (see Figures 2, 3 and Supplementary Figures 1, 2). Comparative nomenclature of the DS or CP in marmoset and mouse is shown in the insets of Figures 1C, 2C, respectively.

### 3.2 Location and gene markers for the caudal CP, CaT∼ and PuC∼ in mice

To explore if any differential gene expression patterns exist between the intermediate CP and caudal CP, we have searched and analyzed gene expression data from the Allen Mouse Brain Atlas^1^ and found some genes that are overall sparsely and densely expressed in the intermediate and caudal CP, respectively. These genes include *Pdyn*, *Otof* (otoferlin) and *Wfs1* (Wolfram syndrome 1 homolog). For example, in the intermediate CP (Figures 2A, B), *Pdyn* is densely expressed in the CPmd (specifically the intermediate/body part of Ca∼: CaB∼) and the medioventral part of the CPlv (specifically the intermediate part of Pu∼: PuI∼) while other parts of the CPlv (DLS) display sparse *Pdyn* expression (Figure 2A). Moving caudally, the sparsely *Pdyn-*expressing region reduces while the densely *Pdyn-*expressing region increases in size (Figures 2B, C). In addition, a very dense and strong *Pdyn*-expressing region (Figure 2B) appears in the most medioventral part of the CPlv, which likely corresponds to the PuPV (PuCv) shown in the Human Brain Reference Atlas (Ding et al., 2016) and in Figures 1B, C. More caudally, where the rostral basolateral amygdaloid nucleus (BL) appears, almost all the dorsal and ventral striatal regions show dense *Pdyn* expression (Figure 2C). The dorsal region, which continues rostrally with the CaB∼ and extends caudally, is labeled as CaT∼ (Figures 2C–H). The ventral region, which is different from the intermediate CP by its overall dense *Pdyn* expression, is labeled as PuC∼ (Figures 2C–H). Still caudally, where the *Pdyn*-expressing central amygdaloid nucleus (Ce) is clearly visible, a *Pdyn*-negative region appears between the Ce and the *Pdyn* expression region (Figure 2D). This *Pdyn*-negative region corresponds roughly to the amygdalostriatal transition area (AStr or ASTA) (Paxinos and Franklin, 2001; Ding et al., 2016). In addition, at the ventral part of the PuC∼, there appear three subregions displaying dense-sparse-dense *Pdyn* expression mediolaterally (Figure 2D). The overall *Pdyn* expression pattern shown in Figure 2D remains toward the end of PuC∼ (Figures 2E–H) although the lateral dense subregion of the PuC∼ disappears at the caudal levels (Figures 2F–H). These three subregions also display differential expressions for other molecular markers (see Valjent and Gangarossa, 2021; Ogata et al., 2022).

Similar expression patterns of the genes *Otof* and *Wfs1* are also observed in the caudal CP except that both genes are also expressed in the AStr, and *Otof* is weakly expressed in the Ce (Supplementary Figures 1, 2). Therefore, based on these gene expression patterns in the CP, a rough boundary between the intermediate CP and caudal CP is placed at the level where the rostral BL appears (e.g., Figure 2C; Supplementary Figures 2A1, A2), meaning the caudal CP (including CaT∼ and PuC∼) is presumed to start at this level (at or after bregma −0.94 mm) in the present study. To facilitate description of CP subregions along the rostral-caudal axis, the CP located rostral to this level and caudal to the midline anterior commissure (ac) is treated as the intermediate CP (including CaB∼ and PuI∼) while all the CP located rostral to the midline anterior commissure is termed rostral CP (including CaH∼ and PuR∼) (see the inset of Figure 2C).

Finally, we have also found a gene marker that is almost selectively expressed in the CaT∼. As shown in Figure 3A, weak *Nts* (neurotensin)-expressing signals in the intermediate CP are mostly localized in the CaB∼ with even weaker expression in the underlying PuI∼. More caudally, *Nts* expression is more clearly visible in the dorsolateral part of the caudal CP (i.e., CaT∼) with almost negative expression in the underlying PuC∼. This pattern starts at the level where the rostral BL clearly appears (Figure 3B) and extends caudally until the end of the caudal CP (Figures 3C–F).

### 3.3 Brain-wide afferent projections of the intermediate and caudal CP

**FIGURE 4:**
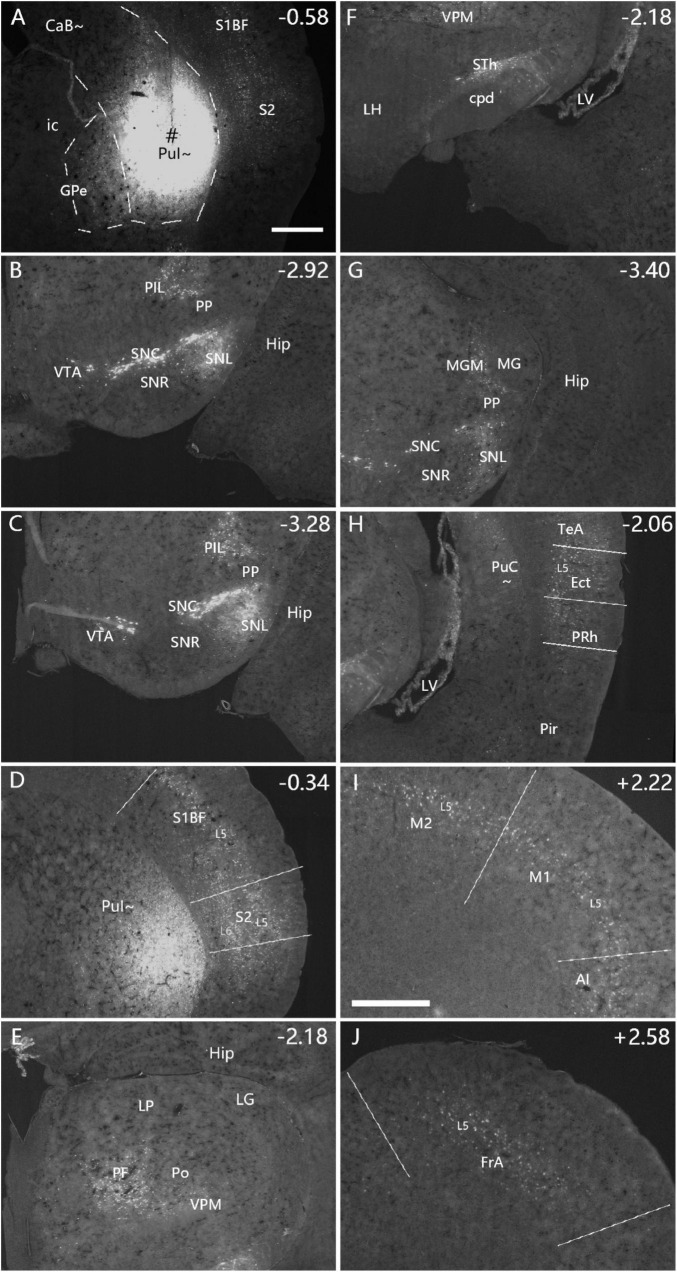
Afferent connections of the PuI∼ revealed with FG. **(A)** An FG injection (#) in the PuI∼. The dashed lines indicate the regional boundaries. **(B–J)** Strongly labeled neurons are found in the SNC **(B)**, SNL **(B)**, VTA **(C)**, PIL **(B,C)**, S2 **(D)**, primary somatosensory cortex [S1BF, S1J, and S1ULp in panel **(D)**], PF **(E)**, VPM **(E,F)**, STh **(F)**, MGM **(G)**, Ect- PRh-TeA **(H)**, M1-M2 **(I)**, AI **(I)** and FrA **(J)**. The text labels for cortical layers are started with an “L” (e.g., L5 for cortical layer 5). Note that layer 5 (L5) of the cortical areas contains most of the labeled neurons. The straight lines in panels **(D,H–J)** indicate approximate borders of the cortical areas. Approximate bregma coordinates are indicated at the top right corner of each panel. Bars: 500 μm in panel **(A)** for panels **(A–H)**; 500 μm in panel **(I)** for panels **(I,J)**.

To reveal and compare brain-wide afferent connections of the PuC∼ and ventral intermediate Pu (PuI∼), we inject the retrograde tracer FG into the PuI∼ (*n* = 5; at bregma −0.58 or −0.70 mm), rostral level of the PuC∼ (*n* = 3; at bregma −1.22) and caudal level of the PuC∼ (*n* = 5; at bregma −1.58 or −1.70 mm). Following an FG injection in the PuI∼ (bregma −0.58; *n* = 3; e.g., Figure 4A), strongly labeled neurons are found in the compact part (SNC) of substantia nigra (SN) (Figure 4B), lateral part of SN (SNL; Figure 4B), ventral tegmental area (VTA; Figure 4C), posterior intralaminar nucleus of thalamus (PIL; Figures 4B, C), secondary somatosensory cortex (S2; Figure 4D), primary somatosensory cortex (S1BF, S1J, and S1ULp; e.g., Figure 4D), parafascicular nucleus (PF; Figure 4E), medial part of ventroposterior medial thalamic nucleus (VPM; Figures 4E, F), subthalamic nucleus (STh; Figure 4F), medial part (MGM) of the medial geniculate nucleus (MG) (Figure 4G), ectorhinal cortex (Ect; Figure 4H), perirhinal cortex (PRh; Figure 4H), temporal association cortex (TeA; Figure 4H), primary motor cortex (M1; Figure 4I), secondary motor cortex (M2; Figure 4I), agranular insular cortex (AI; Figure 4I), frontal association cortex (FrA; Figure 4J). In addition, the rostral part of the basolateral amygdaloid nucleus (BL) and central medial thalamic nucleus also contain FG-labeled neurons. However, FG-labeled neurons are not found in the other part of the MG and the dorsal lateral geniculate nucleus (LG). Additionally, FG injections placed at slightly caudal levels (bregma −0.70 or −0.94 mm; *n* = 2) lead to similar results (e.g., Supplementary Figure 3).

**FIGURE 5:**
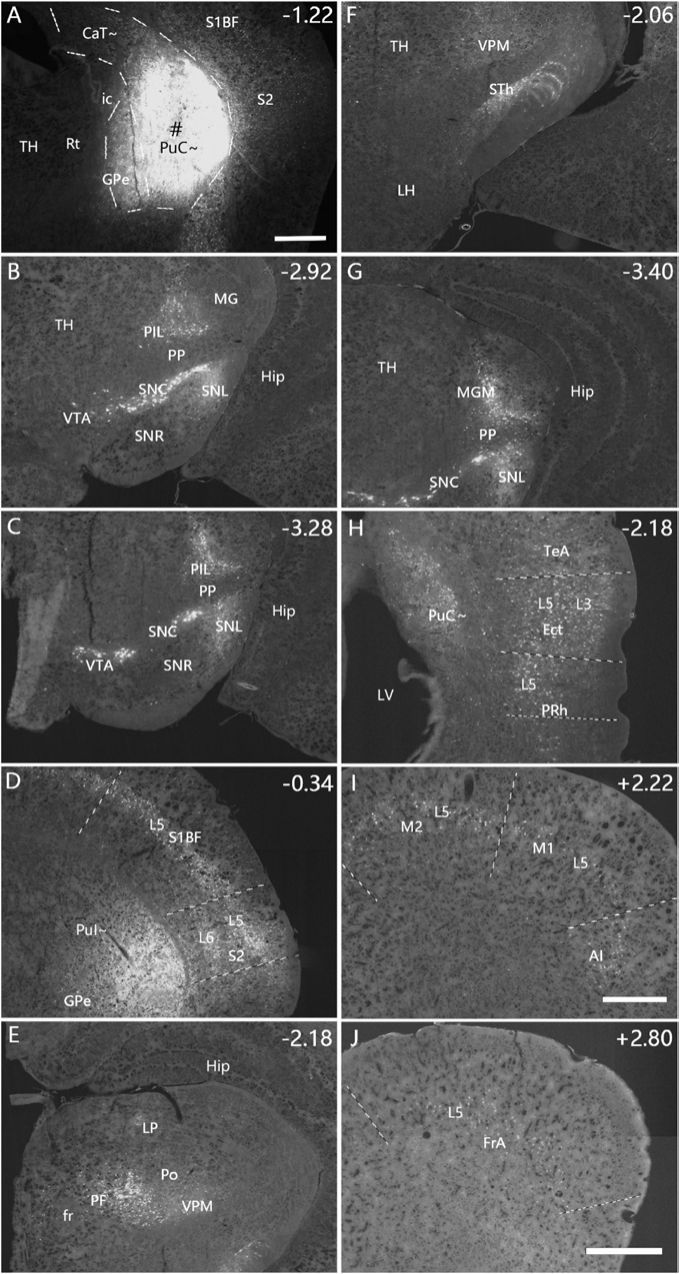
Afferent connections of the rostral PuC∼ revealed with FG. **(A)** An FG injection (#) in the rostral part of PuC∼. The dashed lines indicate the regional boundaries. Three more sections containing the injection site are shown in Supplementary Figures 4A–C. **(B–J)** Labeled neurons are detected in the following regions: SNC **(B)**, SNL **(B)**, VTA **(C)**, PIL **(B,C)**, S2 **(D)**, S1 **(D**), PF **(E)**, VPM **(E)**, STh **(F)**, MGM **(G)**, Ect **(H)**, PRh **(H)**, TeA **(H)**, M1 **(I)**, M2 **(I)**, AI **(I)**, FrA **(J)**, CM, BL and LEC (with fewer labeled neurons; data not shown). The text labels for cortical layers are started with an “L.” Note that layer 5 (L5) of the cortical areas contains most of the labeled neurons. The straight lines in panels **(D,H–J)** indicate approximate borders of the cortical areas. Approximate bregma coordinates are indicated at the top right corner of each panel. Bars: 500 μm in panel **(A)** for panels **(A–G)**; 500 μm in panel **(J)** for panels **(H,J)**; 500 μm in panel **(I)**.

Interestingly, the FG injections placed in the rostral and caudal levels of the PuC∼ (*n* = 8) result in some differential connections. For example, an FG injection restricted in the PuC∼ at the rostral level (*n* = 3; at bregma −1.22 mm, see Figure 5A and Supplementary Figures 4A–C) produces labeled neurons in similar brain regions as in cases with FG injections in the PuI∼ (described above). These similar regions include the SNC (Figure 5B), SNL (Figure 5B), VTA (Figure 5C), PIL (Figures 5B, C), S2 (Figure 5D), S1 (Figure 5D), PF (Figure 5E), VPM (Figure 5E), STh (Figure 5F), MGM (Figure 5G), Ect (Figure 5H), PRh (Figure 5H), TeA (Figure 5H), M1 (Figure 5I), M2 (Figure 5I), AI (Figure 5I), FrA (Figure 5J), CM, BL and lateral entorhinal cortex (LEC) (with fewer labeled neurons; data not shown).

**FIGURE 6:**
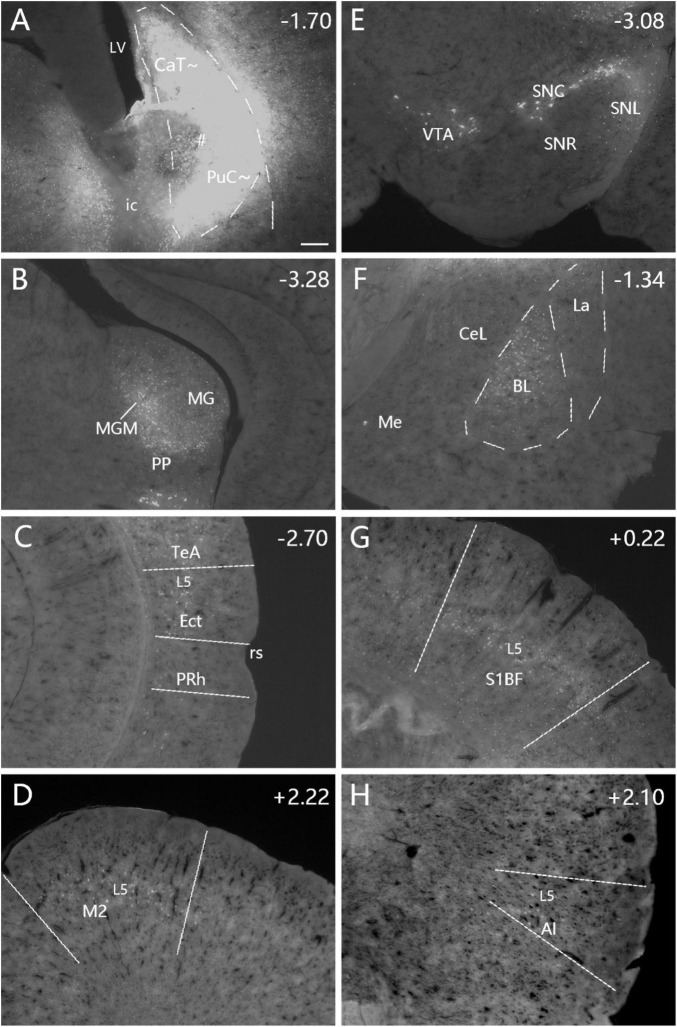
Afferent connections of the caudal CP (CaT∼ + PuC∼) revealed with FG. **(A)** An FG injection (#) involved in both dorsal (CaT∼) and ventral (PuC∼) parts of the caudal CP. **(B–H)** Strongly labeled neurons are observed in the MGM **(B)**, Ect-TeA **(C)**, M2 **(D)**, SNC-VTA-SNL **(E)**, BL **(F)**, S1BF **(G)**, and AI **(H)**. A high magnification view of the labeled neurons in M2 [Panel **(D)]** is shown in Supplementary Figure 4D. Note that layer 5 (L5) of the cortical areas contains most of the labeled neurons. The dashed lines in panels **(A,F)** indicate the regional boundaries. The straight lines in panels **(C,D,G,H)** indicate approximate borders of the cortical areas. Approximate bregma coordinates are indicated at the top right corner of each panel. Bar: 250 μm in panel **(A)** for panels **(A–H)**.

**FIGURE 7:**
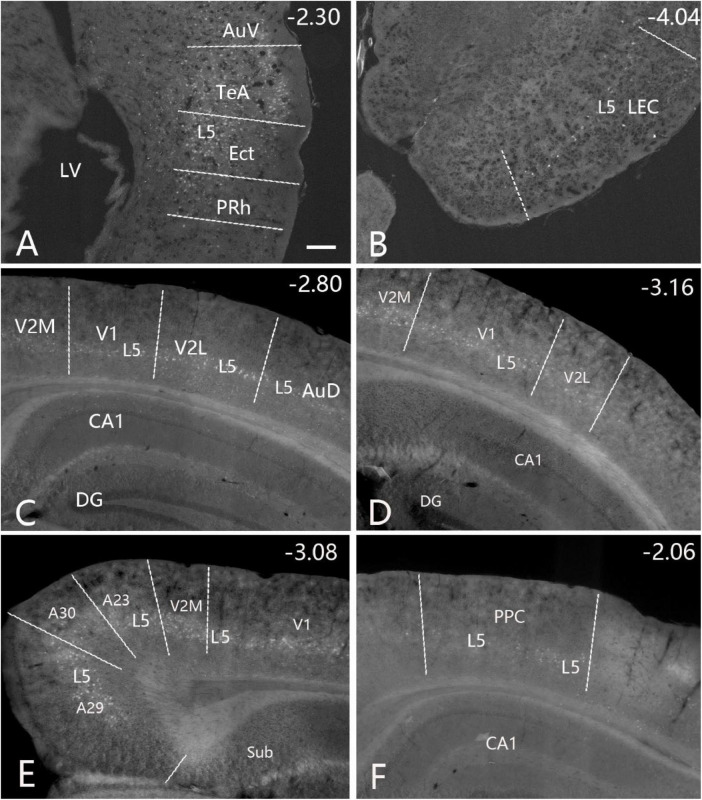
Cortical inputs of the caudal CP (CaT∼ + PuC∼) revealed with FG in the case shown in Figure 6. Many labeled neurons are seen the primary and association auditory cortices [AuV in panel **(A)**; AuD in panel **(C)**], LEC **(B)**, primary (V1) and association (V2M and V2L) visual cortices **(C,D)**, retrosplenial and posterior cingulate cortices [A29, A30, and A23 in panel **(E)**], PPC **(F)** and PoR (data not shown). Note that layer 5 (L5) of the cortical areas contains most of the labeled neurons. The straight lines in panels **(A–F)** indicate approximate borders of the cortical areas. Approximate bregma coordinates are indicated at the top right corner of each panel. Bar: 250 μm in panel **(A)** for panels **(A–F)**.

FG injections at the caudal level of the caudal CP (*n* = 5; at bregma −1.70 or −1.58 mm) are involved in both CaT∼ and PuC∼ (*n* = 2; B −1.70 mm) or only in the PuC∼ (*n* = 3; B −1.58 mm). FG injections in the former cases (e.g., Figure 6A) result in strongly labeled neurons in the SNC, SNL, VTA (Figure 6E), MGM (Figure 6B), BL (Figure 6F), PF, LP (data not shown), Ect, TeA (Figure 6C), S1BF (Figure 6G), M2 (Figure 6D with a high magnification view in Supplementary Figure 4D), AI (Figure 6H), primary and association auditory cortices (Figure 7A for AuV; Figure 7C for AuD), lateral entorhinal cortex (LEC, Figure 7B), primary (V1) and secondary/association (V2M and V2L) visual cortices (Figures 7C, D), retrosplenial (A29 and A30) and posterior cingulate (A23) cortices (Figure 7E), posterior parietal cortex (PPC, Figure 7F) and postrhinal cortex (PoR; data not shown). In addition, it is noted that LG also contains some labeled neurons, likely due to the LG axon fibers passing through the dorsal caudal CP to reach the visual cortex (see Ding, 2024). However, FG injections in the latter cases (only in PuC∼; see Supplementary Figures 5A, G) result in strongly labeled neurons mostly in SNC, SNL, VTA, VPM, MGM, and MGV (Supplementary Figures 5B–F, H–L) with only a few scattered neurons in the BL, PF, LP, Ect, TeA, S1BF, M2, and AI (Supplementary Table 1).

Semi-quantification of FG labeled neurons is performed to score the relative density of the labeled neurons following FG injections into the three rostral-caudal levels of the CP (Supplementary Table 1). Overall, many more labeled neurons are found in the somatosensory-motor (S1, S2, M1, M2, and FrA) and temporal (PRh-Ect-TeA) cortices and in the PF, CM, STh, SNc, and VTA following FG injections into the PuI∼ or rostral level of the PuC∼, compared to the caudal level of the PuC∼. In contrast, more labeled neurons are seen in the Au1, AuD, AuV, MGM, and PIL following the injections into the PuC∼, compared to the PuI∼ (Supplementary Table 1). Interestingly, many more labeled neurons are observed in the S1, S2, M2, AI, LEC, PRh, Ect, and TeA, and in the visual (V1, V2M, and V2L), auditory (Au1, AuD, and AuV), retrosplenial (A29, A30), posterior cingulate (area 23), posterior parietal (PPC) cortices as well as in the amygdala (mainly BL) and PF when the injections are involved in both CaT∼ and PuC∼, compared to the cases, in which the injections are restricted only in the PuC∼ (see the right two columns of Supplementary Table 1). This indicates that the CaT∼ rather than PuC∼ receives many inputs from the somatosensory, motor, parietal, occipital, temporal and cingulate cortices and from the amygdala and PF.

### 3.4 Verification of afferent projections to the PuC∼ using anterograde tracing data

**FIGURE 8:**
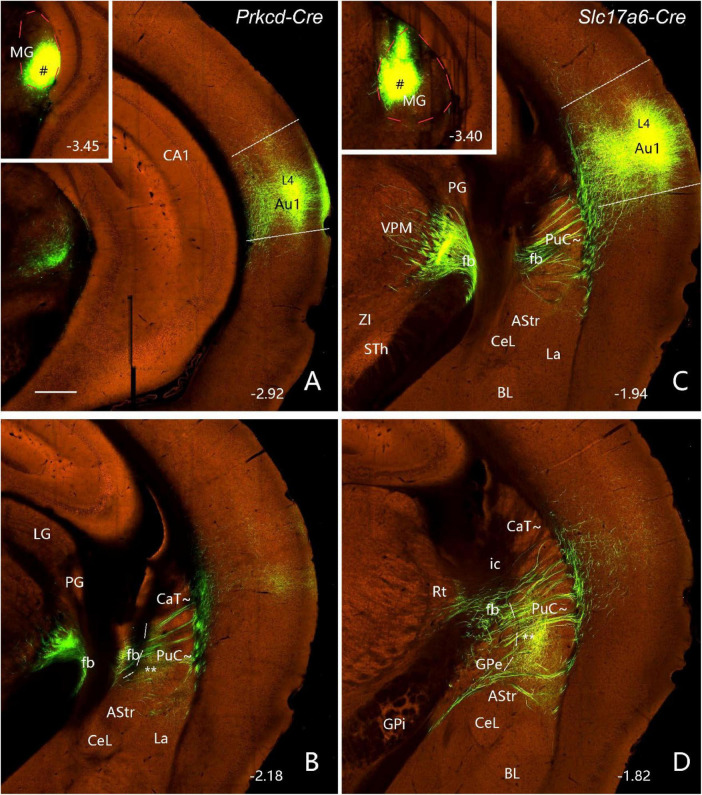
Projections from the MG to the PuC∼ and Au1 (anterograde tracing). **(A,B)** Following an anterograde viral tracer injection into the ventral MG [# in Inset in panel **(A)**] of a *Prkcd-Cre* mouse (ID: 178489574), labeled axon terminals are mainly found in layer 4 (L4) of the Au1 (A) and the medial part of the PuC∼ **(B)**. **(C,D)** Following an anterograde tracer injection into the dorsal MG [# in Inset in panel **(C)**] of a *Slc17a6-Cre* mouse (ID: 305269070), labeled axon terminals are mainly seen in layer 4 (L4) of the Au1 **(C)** and the ventromedial part of the PuC∼ **(D)**. Note that thick fiber bundles (fb) of the MG projections pass through the PuC∼ to reach the Au1 **(B–D)**. The red dashed lines in the insets outline the MG boundaries. The straight lines in panels **(A,C)** indicate approximate borders of Au1. Approximate bregma coordinates are indicated at the bottom right corner of each panel. Bar: 400 μm in panel **(A)** for panels **(A–D)**.

To further confirm the projections from the MG, BL, PoR, and visual and auditory cortices to the caudal CP, related connectional data from the Allen Mouse Brain Connectivity Atlas^3^ are analyzed. Following anterograde viral tracer injections into the ventral and dorsal parts of the MG (insets in Figures 8A, C), densely labeled axon terminals are found in the PuC∼ (Figures 8B, D), in addition to the strong terminal labeling in the primary auditory cortex (Au1; Figures 8A, C). It is noted that the thick fiber bundles of the MG projections pass thorough the PuC∼ to reach the Au1 (Figures 8B–D).

**FIGURE 9:**
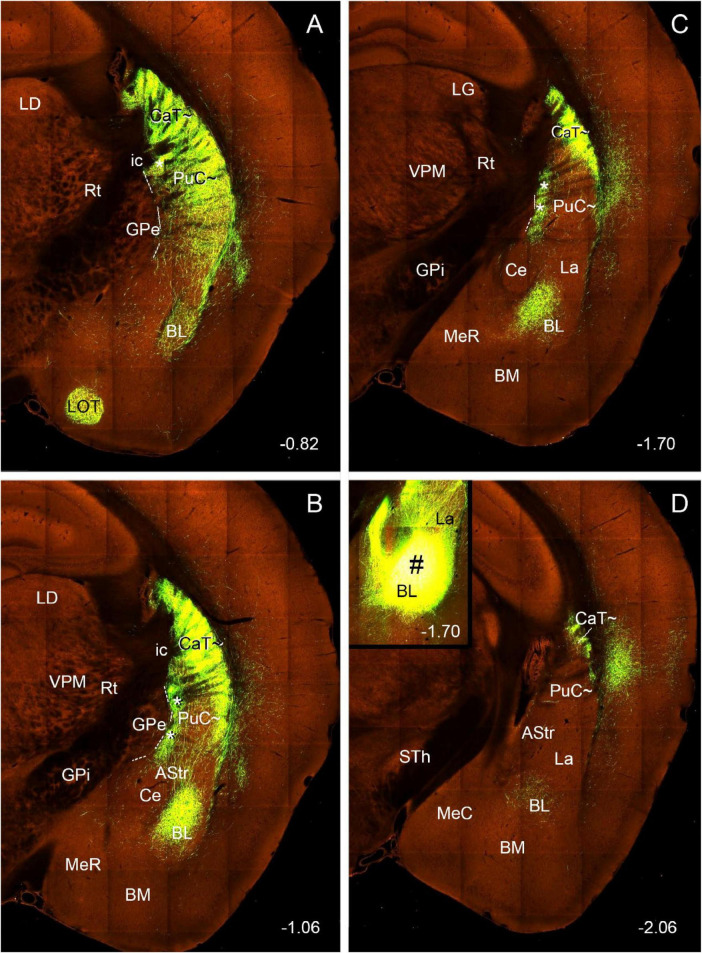
Projections from the BL to the contralateral caudal striatum (anterograde tracing). **(A–D)** Strongly labeled axon terminals are observed in the CaT‘∼ and PuC∼ after a viral tracer injection into the BL [# in Inset in panel (D)] of a wildtype mouse (ID: 113144533). Note that, in the PuC∼, strong terminal labeling is mainly seen in the medial and lateral bands [** in panels **(B,C)**] of the PuC∼. strong terminal labeling is also seen in the Ca∼ (CaH∼ and CaB∼) and Pu∼ (PuR∼ and PuI∼) of the rostral and intermediate CP (not shown), LOT (A) and all parts of the BL **(A–D)**. Note that much stronger terminal labeling is found in the ipsilateral striatum. Approximate bregma coordinates are indicated at the bottom right corner of each panel. Bar: 560 μm in panel **(A)** for panels **(A–D)**.

Interestingly, the amygdala (mainly BL) projects very heavily to the CP along the rostral-caudal axis in both hemispheres with the ipsilateral hemisphere (data not shown) showing much stronger terminal labeling than the contralateral one (Figure 9). Both ipsilaterally and contralaterally, strongly labeled axon terminals are observed in the dorsolateral part (see Figures 9A–D) of the caudal CP (mostly within the CaT∼ identified with *Nts* expression; see Figure 3) after the viral tracers are injected into the BL (inset in Figure 9D). Dense terminal labeling is also seen in the medial part of the PuC∼ (** in Figures 9B, C) and other parts of the BL (Figures 9A–D). It should be mentioned that very heavy terminal labeling is seen in the mediodorsal CP (part of Ca∼) and medioventral parts (part of Pu∼) of the rostral and intermediate CP, NAC-OT and the nucleus of lateral olfactory tract (LOT; Figure 9A). Additionally, the anterograde tracer injections into the lateral PF also led to labeled axon terminals mostly in the CaT∼ and dorsal part of PuC∼ (see Supplementary Figure 6) and this is consistent with the FG tracing results described in the above section.

**FIGURE 10:**
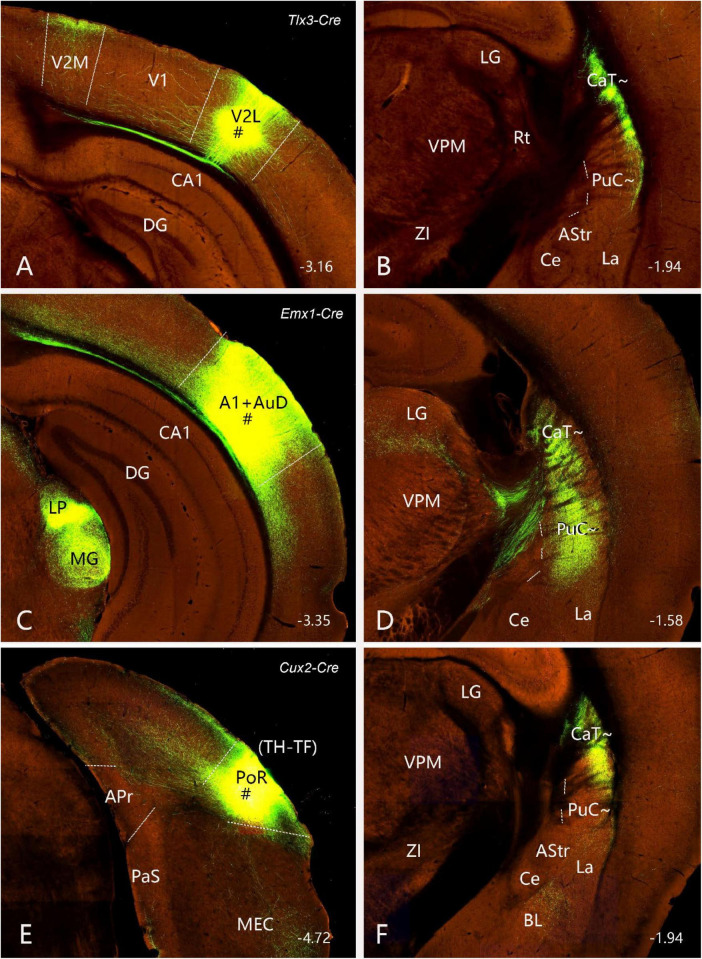
Projections from the cerebral cortex to the CaT∼ and PuC∼ (anterograde tracing). **(A,B)** Projections from the lateral association visual cortex (with injection # in V2L) **(A)** to the CaT∼ **(B)** in a *Tlx3-Cre* mouse (ID: 297231636). **(C,D)** Projections from the primary and association auditory cortex (with injection # in A1 + AuD) **(C)** to the CaT∼ and PuC∼ **(D)** in an *Emx1-Cre* mouse (ID: 579203888). **(E,F)** Projections from the postrhinal cortex [with injection # in PoR [panel **(E)**; equal to areas TH + TF in primate brains] to the CaT∼ **(F)** in a *Cux2-Cre* muse (ID: 287953929). It is noted that labeled axon terminals in these cases strongly target the CaT∼ **(B,D,F)**, and the auditory cortical afferents also heavily terminate in the PuC∼ **(D)**. Note that *Tlx3-Cre* is selectively expressed in cortical layer 5, while *Emx1-Cre* expresses in excitatory neurons in all cortical layers (including layer 5); *Cux2-Cre* is strongly expressed in layers 3–4 with sparse expression in cortical layer 5 (see Hu et al., 2020; Lu et al., 2020). The straight lines in panels **(A,C,E)** indicate approximate borders of cortical areas. Approximate bregma coordinates are indicated at the bottom right corner of each panel. Bar: 400 μm in panel **(A)** for panels **(A–F).**

As described above, FG injections in both intermediate and caudal CP lead to labeled neurons in layer 5 of most cortical areas with exception in the Prh-Ect-PoR regions, where FG labeled neurons are also found in layers 2–4 (see Figures 4–7). To confirm these cortico-striatal projections using Cre-dependent anterograde tracing experiments, we choose some available Cre-line mice with Cre expression in layer 5 (e.g., *Tlx3-Cre)*, layers 2–6 (e.g., *Emx1-Cre)*, and layers 2–4 (e.g., Cux2*-Cre)* from the Allen Mouse Connectivity Atlas. The results show that cortical afferents of the caudal CP from the lateral association visual cortex (Figure 10A, from a *Tlx3-Cre* case), primary and association auditory cortex (Figure 10B, from a *Emx1-Cre* case), PoR (Figure 10C, from a *Cux2-Cre* case), M2, S1, S2, and PPC (data not shown) all terminate mostly in the dorsolateral part of caudal CP (i.e., CaT∼, Figures 10D–F) with the auditory cortical afferents also in the PuC∼ (Figure 10E). It is also noted that posterior cingulate area 23 (A23) in rodents has been shown to project to the regions corresponding to the rostral Ca (CaH∼ and CaB∼) and caudal Ca (CaT∼) [labeled as CPu-p in Xiang et al. (2023)].

Finally, following the viral tracer injections into the VPM (Supplementary Figure 7A), labeled axon terminals are observed mainly in the PuC∼ (Supplementary Figure 7B) and caudal part of the PuI∼. In addition, many strongly labeled axon fiber bundles (fb; with big diameters) are also seen passing through the PuC∼ (Supplementary Figure 7B). At high magnification, the labeled axon terminals display obvious varicosities while the fiber bundles do not (Supplementary Figure 7C).

In summary, these findings from anterograde tracing confirm the results from FG retrograde tracing described in above section and further reveal the detailed locations and extent of the axon terminal fields.

### 3.5 Efferent projections of the PuI∼ and PuC∼

**FIGURE 11:**
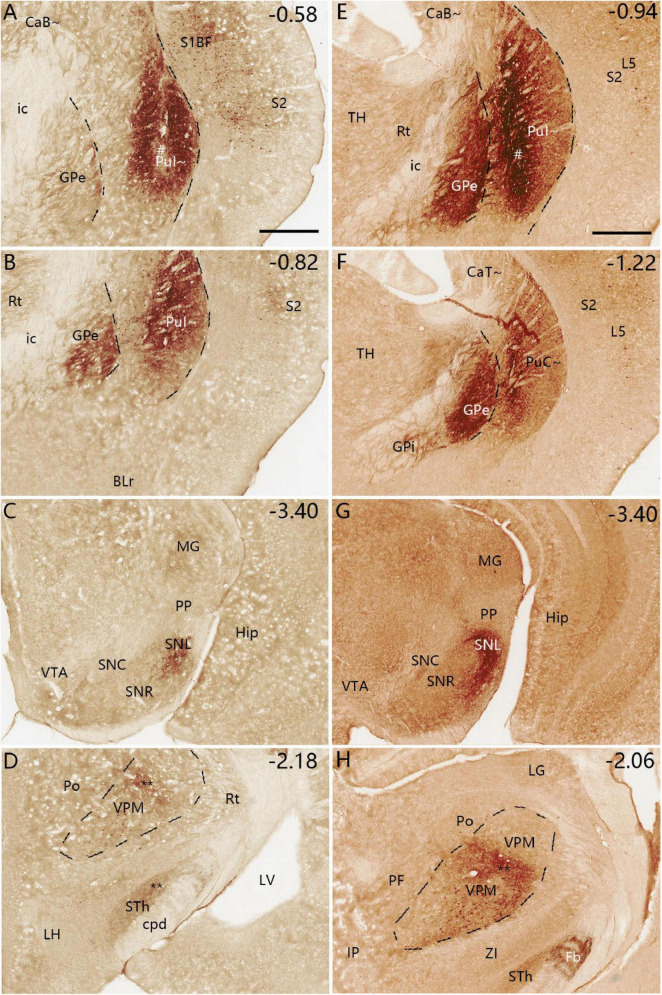
Efferent projections of the PuI∼ revealed with BDA. **(A–D)** One BDA injection into the rostral PuI∼ [# in panel **(A)**] leads to labeled axon terminals in the ventral GPe **(B)**, SNL **(C)** and middle VPM **(D)**. Faint BDA labeling is detected in the lateral STh **(D)**. Three more sections containing the injection site are shown in Supplementary Figures 8A–C. **(E–H)** Another BDA injection into the caudal PuI∼ [# in panel **(E)**] produces labeled axon terminals in the ventral GPe **(F)**, SNL **(G)**, and middle VPM **(H)**. Weak labeling is detected in the lateral STh **(H)** and lateral GPi **(F)**. A high magnification view of the labeled axon terminals in VPM [Panel **(H)**] is shown in Supplementary Figures 8D. Note that some BDA-labeled cell bodies are seen in S1, S2 and VPM **(A,B,D,E,F,H)**. Dashed lines indicate regional boundaries. Approximate bregma coordinates are indicated at the top right corner of each panel. Bars: 500 μm in panel **(A)** for panels **(A–D)**; 500 μm in panel **(E)** for panels **(E–H)**.

**FIGURE 12:**
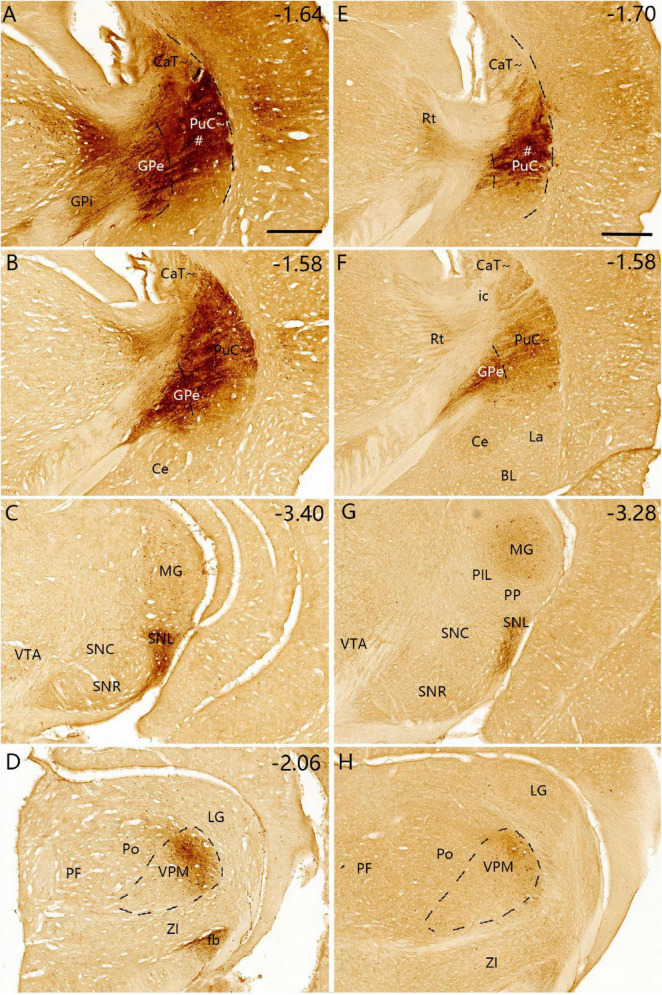
Efferent projections of the PuC∼ revealed with BDA. **(A–D)** One BDA injection into the middle PuC∼ [# in panel **(A)**] leads to labeled axon terminals in the ventral GPe **(B)**, SNL **(C)** and lateral VPM **(D)**. **(E–H)** Another BDA injection into the most caudal PuC∼ [# in panel **(E)**] produces labeled axon terminals in the ventral GPe **(F)**, SNL **(G)** and lateral VPM **(H)**. BDA-labeled cell bodies are found in S1, S2, MG and VPM **(A–D,G,H)**, consistent with the FG tracing results. A high magnification view of the labeled axon terminals in SNL (Panel **(G)**] is shown in Supplementary Figure 8E. Dashed lines indicate regional boundaries. Approximate bregma coordinates are indicated at the top right corner of each panel. Bars: 500 μm in panel **(A)** for panels **(A–D)**; 500 μm in panel **(E)** for panels **(E–H)**.

To reveal and compare brain-wide efferent projections of the PuI∼ and PuC∼, the anterograde tracer BDA is injected into the PuI∼ (*n* = 4; bregma: −0.58 or −0.82 mm) and the PuC∼ (*n* = 2; Bregma: −1.58 or −1.70 mm) (e.g., Figures 11, 12). When BDA injections are placed into the PuI∼ (Figures 11A, E and Supplementary Figures 8A–C), labeled axon terminals are mainly found in the ventral GPe (Figures 11B, F) and SNL (Figures 11C, G) with weak (** in Figure 11D) and moderate (** in Figure 11H) labeling in middle VPM. Weak terminal labeling is also seen in the lateral STh (Figure 11D) and GPi (Figure 11F). Higher magnification view of the labeled terminals in the VPM in Figure 11H is shown in Supplementray Figure 8D. In addition, BDA-labeled cell bodies are visible in S1, S2 and VPM (Figures 11A, B, D–F, H).

Following BDA injections into the PuC∼ (*n* = 2; Figures 12A, E), labeled axon terminals are mainly detected in the caudal GPe (Figures 12B, F), SNL (Figures 12C, G) and lateral VPM (Figures 12D, H). Higher magnification view of the labeled terminals in the SNL in Figure 12G is shown in Supplementary Figure 8E. The varicosities of the axon terminals can be clearly seen in the SNL and GPi in viral tracing cases (e.g., Supplementary Figures 8F, G). S1, S2, MG, and VPM also contain BDA-labeled cell bodies.

To test the possibility that labeled axon terminals in VPM may result from tracer leaking into adjacent GPe from injection site in the PuC∼ (e.g., Figures 11E, 12A), we have examined the cases with viral tracer injections restricted in the GPe. The results show that mouse GPe strongly projects to the PF, STh and SNR but not to VPM (Supplementary Figure 9). However, the possibility could not be excluded that BDA might be taken up by some of the descending cortical fibers that traverse the striatum and results in some terminal labeling in the VPM. Therefore, more experiments are needed in future to determine if striatal-VPM projections exist.

### 3.6 Verification of efferent projections of the PuI∼ and CaT∼

**FIGURE 13:**
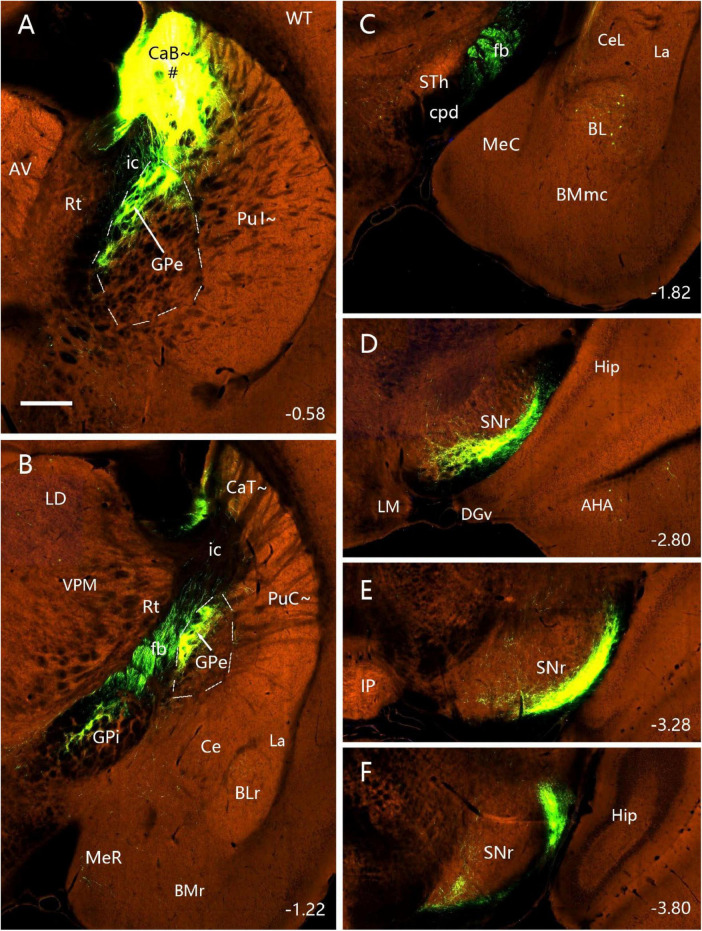
Efferent projections of the CaB∼ (anterograde tracing). **(A–F)** The anterograde tracer injection [# in panel (A)] in a wild-type mouse (ID: 127762867) leads to strong terminal labeling in the dorsal GPe **(A,B)**, dorsal GPi **(B)**, ventrolateral SNR and SNL **(D–F)**. Note that some retrogradely labeled neurons are seen in the BL **(C)** and no terminal labeling in the STh **(C)**. Dashed lines outline the boundaries of the GPe. Approximate bregma coordinates are indicated at the bottom right corner of each panel. Bar: 400 μm in panel **(A)** for panels **(A–F)**.

**FIGURE 14:**
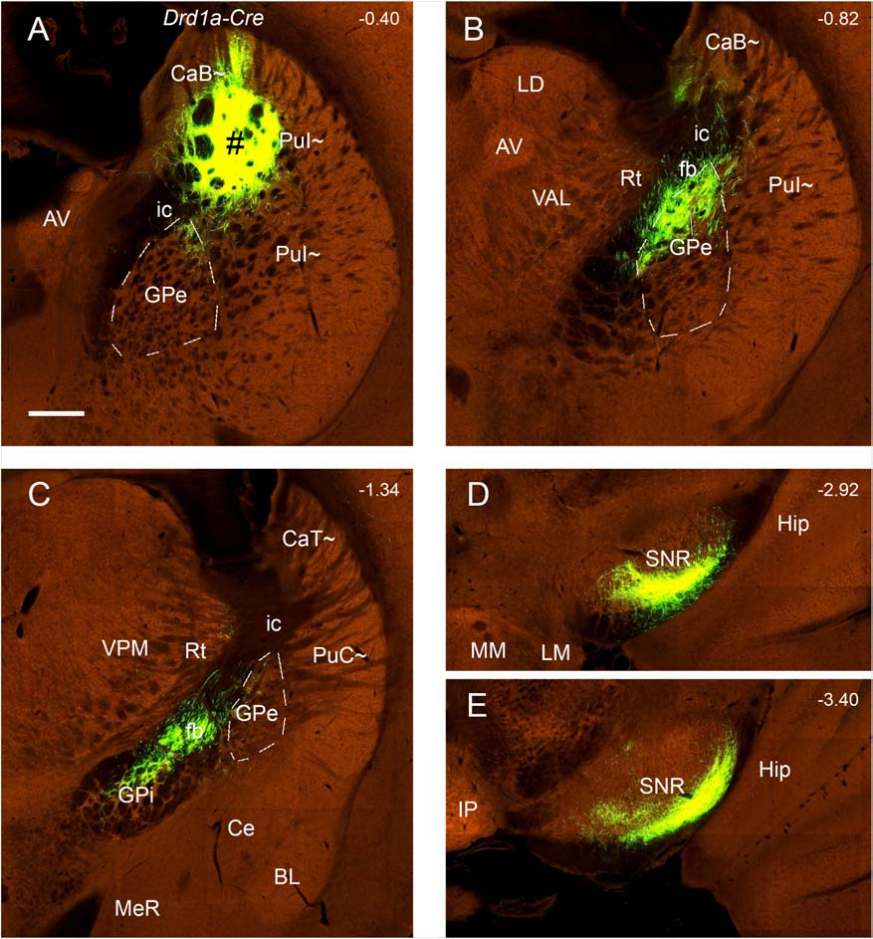
Efferent projections of the CaB∼ and dorsal PuI∼ (anterograde tracing). **(A–E)** The anterograde tracer injection [# in panel **(A)**] in a *Drd1a-Cre* mouse (ID:159941339) leads to strong terminal labeling in the dorsal GPe **(B)**, dorsal GPi **(C)** and ventrolateral SNR **(D,E)**. Note that these results are very similar to those shown in the wild-type mouse (Figure 13). Dashed lines outline the boundaries of the GPe. Approximate bregma coordinates are indicated at the top right corner of each panel. Bar: 400 μm in panel **(A)** for panels **(A–E)**.

To reveal and confirm the main efferent projections of the PuI∼ and CaT∼, the Allen Mouse Brain Connectivity atlas^3^ is searched, and three wildtype, two Plxnd1-Cre and one Drd1a-cre mice with anterograde tracer injections in the dorsal PuI∼ and CaT∼ are analyzed. In all six cases, labeled axon terminals are found in the dorsal GPe, dorsal GPi and ventrolateral SNR with no terminal labeling in the STh (e.g., Figures 13A–F for the wild-type case and Figures 14A–E for the Drd1a-cre case). In addition, some labeled neurons are seen in the BL (e.g., Figure 13C).

### 3.7 A unique efferent projection pattern of Drd2-expressing neurons in all CP

**FIGURE 15:**
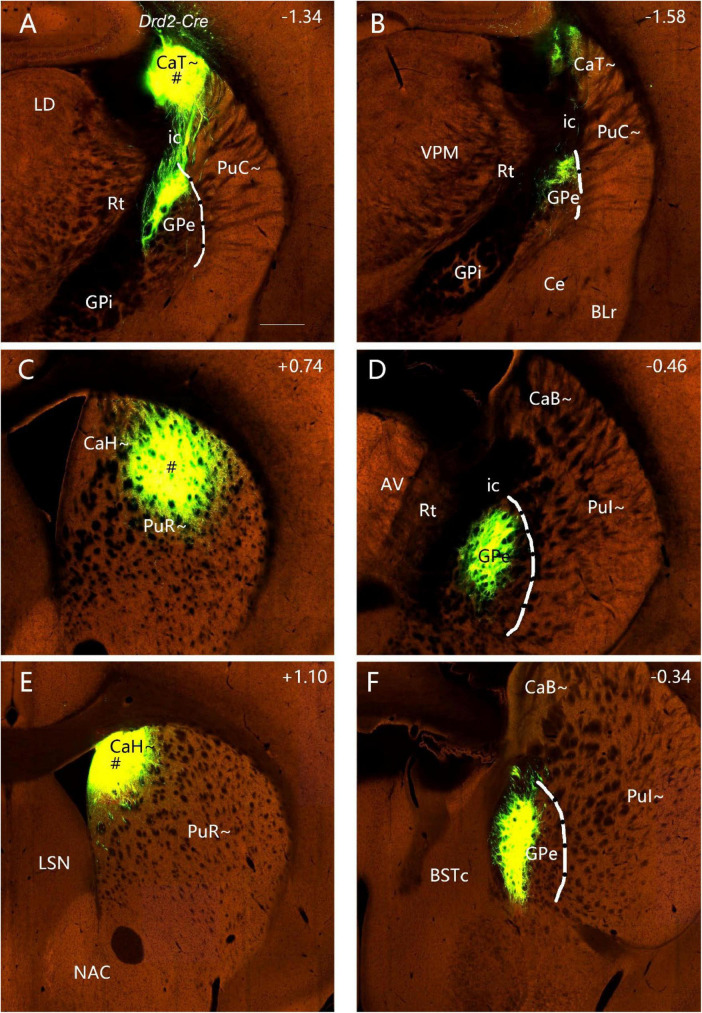
Efferent projections of the striatum in *Drd2-Cre* mice (anterograde tracing). **(A,B)** The anterograde tracer injection into the CaT∼ [# in panel **(A)**] (ID: 160540013) leads to strong terminal labeling only in the dorsal GPe **(B)**. **(C,D)** The tracer injection into the dorsal PuR∼ [# in panel **(C)**] (ID: 158019342) also produces strong terminal labeling only in the rostral dorsal GPe **(D)**. **(E,F)** The tracer injection into the CaH∼ [# in panel **(E)**] (ID:127224133) leads to strong terminal labeling only in the rostral dorsomedial GPe **(F)**. In all these and other Drd2-Cre mice (data not shown), no axon terminal labeling is detected in the GPi and SNR. Dashed lines indicate the border between GPe and Pu∼ (PuC∼ and PuI∼). Approximate bregma coordinates are indicated at the top right corner of each panel. Bar: 400 μm in panel **(A)** for panels **(A–F)**.

To explore if the rostral, intermediate and caudal CP in certain Cre-line mice display unique cell-type specific efferent projections, we have examined available four Drd1a-Cre_EY262 mice, one Slc32a1-IRES-Cre mouse, one Slc18a2-Cre_OZ14 mouse, one Gad2-IRES-Cre mouse, ten Drd2-Cre_ER44 mice, two Penk-IRES2-Cre_neo mice and one Efr3a-Cre_No108 mouse from the Allen Mouse Brain Connectivity atlas. Like in the wild-type mice, the anterograde tracer injections into many parts of the CP (including caudal CP) of the Drd1a-Cre, Slc23a1-Cre, Slc18a2-Cre and Gad2-Cre result in labeled axon terminals in the common target regions GPe, GPi and SN with rough topographical distribution (see Figures 14A–E for a Drd1a-Cre mouse; Supplementary Figures 10A–C for a Gad2-Cre mouse). However, in all Drd2-Cre (*n=9*) and Penk-Cre (*n=2*) mice with injections in many parts of CP (including caudal CP), labeled axon terminals are only observed in the GPe with rough topography (see example in Figures 15A–F) with no terminal labeling in other regions including the GPi and SN. Additionally, in the Efr3a-Cre mouse with a tracer injection in the CP (CaT∼), axon terminal labeling is mainly seen in the SN with weak labeling in the GPe (Supplementary Figures 10D–F).

## 4 Discussion

The CP (DS) of rats and mice consists of a single mass of cells and is not divided into the Ca∼ and Pu∼ because of discrete fibers of the internal capsule in the CP. Classical embryological studies suggested the hypothesis that the neostriatum develops as a part of the telencephalic roof or pallium (Kallen, 1962). Many previous studies have established that different parts of the CP receive afferent inputs from different cortical regions and send their outputs to the cortex via the thalamus (e.g., Parent and Hazrati, 1995; Haber, 2003). The striatum sorts contextual, motor, and rewards information from its two major excitatory input sources (the cortex and the thalamus) into distinct downstream pathways (Hunnicutt et al., 2016; Mandelbaum et al., 2019; Foster et al., 2021). Understanding precise circuitry among the cortex, thalamus, and striatum is crucial to dissect mechanistically how these structures coordinate related functions. In this study, we have investigated brain-wide afferent and efferent projections of the less-studied caudal CP, which appears to contain the CaT∼ (dorsal part) and PuC∼ (ventral part). We have also revealed and compared the differential connections of the CaT∼ and PuC∼. Finally, using Cre-dependent anterograde tracing in Cre-line mice, we have further confirmed that Drd2- and Penk-expressing neurons in the CP (including the CaT∼ and PuC∼) exclusively target the GPe while Drd1a-expressing neurons (and some others) in the CP innervate GPe, GPi and SN.

### 4.1 Identification and localization of mouse equivalent of the primate caudate tail

In primates including humans, the Ca and Pu are clearly separated by the big dense fiber bundle, internal capsule, and the Ca can be subdivided into the head, body and tail with the tail extending to the ventral aspect of the CP and adjoining the PuCv and AStr (Yeterian and van Hoesen, 1978; Van Hoesen et al., 1981; Saint-Cyr et al., 1990; Cho et al., 2013; Ding et al., 2016, also see Figure 1). In rats and mice, however, the Ca∼ and Pu∼ are mostly merged since the fibers in internal capsules are loosely packed in the CP and thus are not able to clearly separate the Ca∼ from the Pu∼ (see Figure 2). Furthermore, it is not clear whether the CaT∼ exists in rats and mice.

In the present study, we have provided clear evidence in gene expression and connectivity for the identification and localization of mouse CaT∼. First, all Ca (including CaT) in primates (e.g., marmoset) shows strong and overall homogenous *Pdyn* expression while typical Pu (including PuC) displays overall weak *Pdyn* expression (see Figure 1). Similarly, in mice, we find that *Pdyn* is strongly expressed in the mediodorsal part of the CP (akin to the Ca in primates) and this region extends caudally along the dorsolateral part of the CP (see Figure 2), suggesting that this caudal extension of the Ca likely corresponds to the CaT in primates (thus termed CAT∼). Since the ventral part of the caudal CP (PuC∼) also displays strong *Pdyn* expression, it is difficult to identify the border between the CaT∼ and PuC∼. Fortunately, we have discovered another gene *Nts*, which is expressed in the Ca∼ with stronger expression in CaT∼, compared to the PuC∼, which displays no or faint *Nts* expression (see Figure 3). This *Nts* expression pattern makes the shape and size of the CaT∼ stand out (see Figures 3B–F). Specifically, the CaT∼ in the mice occupies the dorsolateral part of the caudal CP (see Figures 3B–F), and this location is further confirmed with the location of the labeled axon terminal field originating from the BL (Figure 9), PF (Supplementary Figure 6), lateral visual cortex (Figures 10A, B), PoR (Figures 10E, F) and S1–S2 (data not shown). In all these cases, the resulted axon terminals are mostly restricted in the CaT∼ region identified with the *Nts* expression.

Second, the main afferent inputs of the mouse CaT∼ revealed in the preset study and of the primate CaT reported in the literature are similar and comparable. Specifically, the monkey CaT receives strong inputs from the amygdala (mainly BL) and many visual and temporal cortices including areas TE, TH-TF and V3–V4 (Yeterian and van Hoesen, 1978; Van Hoesen et al., 1981; Saint-Cyr et al., 1990; Cho et al., 2013; Griggs et al., 2017). Consistently, in the present study, when the FG injections are involved in the CaT∼, many labeled neurons are detected in the BL, PF and the visual, auditory, retrosplenial and posterior parietal cortices (e.g., Figures 6, 7 and Supplementary Table 1). These connections are further confirmed with anterograde tracing experiments (see Figures 9, 10 and Supplementary Figure 6).

Therefore, all these findings support the identification and localization of the CaT∼ in mice (and likely also in rats). The convergent projections to the CaT (CaT∼) from multimodal regions also suggest that CaT (Cat∼) is a hub for the integration of multimodal sensory, motor and limbic information (Valjent and Gangarossa, 2021; Lee et al., 2023; the present study).

### 4.2 Comparison with previous studies on the afferent projections of the caudal CP

In previous studies, projections from the SNL of the cat (Kaelber and Afifi, 1979), SNC of the rat (Tulloch et al., 1978) and VTA of the mouse (Mizoguchi et al., 2021) to the Pu∼ (mostly PuR∼ and PuI∼) were reported. Our experiments on the caudal CP in the mice have resulted in similar results regarding the input source regions such as SNC, SNL (∼lateral SNR) and VTA. The retrograde tracer injections into the caudal striatum of the rats lead to labeled cells in the PF, VPM and central medial nucleus (Elena Erro et al., 2002). Lesions of the cat centromedian nucleus (CM) result in severe degenerated axonal terminals in the PuR∼ and PuI∼ (Kuroda et al., 1975). Labeled axonal terminals are also discerned in the PuI∼ when the anterograde tracers are placed in the MGM and MGV of the rats (Veening et al., 1980; LeDoux et al., 1985, 1991) and in the PIL-PP of the rats (Cai et al., 2024). These connections are also confirmed in mice in the present study. The projections from the cortical regions to the tail of striatum (∼caudal CP in this study) have been reported in rats and these regions include M1, M2, AI, S1, S2, V2M, V2L, A1, AuD, AuV, PPC, LEC, PRh, and Ect (Jiang and Kim, 2018). Similar afferent connections of the caudal striatum are also reported in mice (Pan et al., 2010). However, these studies do not subdivide the caudal CP into dorsal (CaT∼) and ventral (PuC∼) parts. The present study has revealed that the projections from M1, M2, S1, S2, V1, V2M, V2L, PoR, PPC, BL, PF, and LP-Pul mostly terminate in CaT‘∼ while those from the MG, PIL and VPM mainly innervate the PuC∼. Importantly, the projections from the SNL, LEC, A1 and A2 (AuD and AuV) terminate both in CaT∼ and PuC∼.

In summary, our results show that the afferent projections from multimodal cortical regions in the somatosensory, visual, auditory, posterior cingulate and parietal cortices and from the association thalamic nuclei such as PF and LP converge mainly on the CaT∼ (dorsal part of the caudal CP) rather than on the PuC∼ (ventral part of the caudal CP). Interestingly, heavy inputs from the amygdala (mainly BL), which is a hub for emotion procession, and the SNL, which is important for rewards and value-coding, also converge on the CaT∼.

### 4.3 Comparison with previous studies on the efferent projections of the caudal CP

The major outputs of the caudal CP target the same output structures such as GPe, GPi and SN but with rough topographical terminal locations within the same structures, as other CP regions do. Specifically, the PuC∼ projects to the ventral GPe and GPi, and lateral SNr (SNL). In contrast, the overlying CaT∼ innervates the dorsal GPe and GPi and ventrolateral SNr. Interestingly, the PuC∼ but not the CaT∼ appears to project to the VPM. These findings are also generally consistent with previous studies on the rostral-intermediate striatum. For instance, the retrograde tracer injections into the primate GPe and GPi lead to clustered labeled cells in the Pu (Flaherty and Graybiel, 1993). Lesion in the caudal CP of the rats leads to degenerated axon fibers in the SNL while lesion in the rostral CP produces degenerated fibers in the SNc and SNr (Kohno et al., 1984). Additionally, the injections of FG and BDA into the rat STh have revealed bidirectional connections of the Ca∼ and Pu∼ with STh (Cavdar et al., 2018). These connections are also consistent with our results in mice although they are relatively weak compared to other connections.

### 4.4 Comparison of the projections from the PuC∼ and PuR∼

Many previous studies have reported the connections of the rostral-intermediate striatum while the present study focuses on the caudal striatum (from bregma −1.06 mm to the caudal end). It would be helpful to compare the connections of the PuC∼ and more rostrally located Pu∼ (PuR∼ and PuI∼) to evaluate possible differences along the rostral-caudal axis. The efferent connectional patterns of these caudal-rostral Pu∼ are overall similar. All exhibit strong projections to the GPe, GPi and SN with rough topographic distribution in the same target regions. Specifically, the PuC∼ projects mainly to caudal ventral GPe and GPi, and caudal ventrolateral SN (e g., Figure 12) while the PuR∼ (rostral to the anterior commissure) projects mainly to the rostral ventral GPe and GPi, and rostral ventromedial SN; the PuI∼ (from the commissure to the level at bregma −0.94 mm) innervates mainly the regions in between (Hintiryan et al., 2016). Compared to ventral Pu∼, dorsal Pu∼ and Ca∼ project to the subregions in the GPe and GPi dorsal to those from the ventral Pu∼, and to the subregions in the SN ventral to those from the ventral Pu∼. Finally, one distinct target of the PuC∼ and PuR∼ is the VPM. The PuC∼ rather than the PuR∼ appears to project to the VPM (e.g., Figures 11, 12).

As for the afferents to the caudal and rostral CP, the difference in the strength of many afferent connections is obvious between the caudal and rostral CP. For instance, the rostral Ca∼ receives stronger projections from the prefrontal (PL, IL, OrB), motor (M1, M2), and primary visual (V1) cortices compared to the caudal Ca∼ (Pan et al., 2010; Griggs et al., 2017; Jiang and Kim, 2018; Miyamoto et al., 2018). Rostral Ca also receives inputs from the medial temporal cortices in monkeys (Choi et al., 2017). In contrast, S1BF, auditory (A1, A2) and temporal association (TeA and PoR) cortices projects strongly to the caudal CP than to rostral CP (McGeorge and Faull, 1989; Pan et al., 2010; the present study). Additionally, the PPC, Ect and V2 project strongly to both rostral and caudal Ca∼∼ (Allen connectivity data). For subcortical inputs, the PuC∼ but not the PuR∼ and PuI∼ receive inputs from the VPM and MG (see Figures 8, 11, 12 and Supplementary Figure 7). Compared to caudal Ca∼, rostral Ca∼ receives much stronger inputs from the PF (Allen connectivity data).

### 4.5 Functional relevance of the connections of the caudal CP

Previous studies in primates indicate that the caudal striatum mainly receives afferent projections from many higher order of visual cortices in the parietal, occipital and temporal cortex (Saint-Cyr et al., 1990). The caudal striatal region is primarily involved in visual processing, such as object recognition, visual saliency, and habitual behaviors of visual value judgment (Kim and Hikosaka, 2013, 2015). The caudal striatum in rats (so-called tail of the striatum) also receives inputs from brain regions related to value judgment, such as the SN (Tobler et al., 2005; Jiang and Kim, 2018) and may play a major role in the long-term value memory encoding of habitual glances at valuable objects, while the rostral striatum is not involved in this process (Jiang and Kim, 2018). Consistently, the present study reveals that the caudal striatum of the mice receives afferents from the SN and primary and secondary visual cortices. Additionally, the inputs from multiple sensory cortices innervate (converge in) the caudal striatum in mice, and these inputs include those from visual, auditory, and somatosensory cortices. The caudal striatum also receives inputs from multiple sensory (e.g., VPM, LP, PIL and MG) and motor (e.g., PF) thalamic regions. All these suggest that the caudal striatum is anatomically capable of linking sensory stimulus with motor responses in habitual behaviors. Consistently, the dopaminergic axon terminals in the caudal striatum respond to different types of sensory stimulus, including visual, auditory, and somatosensory (Menegas et al., 2018). All these correlations appear to be associated mainly with the Ca∼, which receives multimodal inputs, as revealed in the present study.

In addition to the dorsally located CaT, the caudal striatum in rodents also contains the ventrally located caudal Pu (PuC∼), which receives its inputs mainly from auditory structures such as the PIL, MGM, A1 (Au1), A2 (AuD and AuV) and the truck and limb representation fields of S1. Given that these distinct subcircuits originate from distinct brain regions, they may exhibit functional differences (Haber, 2016; Ni et al., 2021). The similarities and differences in the whole-brain projection patterns of the CP might provide novel insights into the distinct functions of the various striatal subcircuits.

The behavioral characteristics of addiction likely reflect an imbalance between goal-directed behavior and habit formation driven by stimulus (Ersche et al., 2021). For adolescents suffering from Internet game addiction, the functional connectivity between the dorsal Pu∼ and S1 are more robust (Hong et al., 2015). There is mounting evidence suggesting that rapid goal-directed learning primarily involves the DMS (Ca∼), while slower habit acquisition, which is insensitive to alterations in the rewards value of the outcome, involves the Pu∼ (Yin and Knowlton, 2006; Balleine et al., 2007). As habitual behaviors form, the control point by the striatum shifts from the Ca∼ to Pu∼. The enhanced functional connections between the dorsal Pu∼ and S1 may promote the neural binding of game-related sensory stimuli and habitual behavioral patterns, ultimately resulting in the maintenance and relapse of addiction. It is speculated that the projections between Pu∼ and S1 are related to addiction.

The clinical manifestations of Parkinson’s disease have stage-specific characteristics. In the early stage of the disease, patients mainly present with motor-related symptoms (such as bradykinesia, muscle rigidity, etc.), which are related to the dysfunction of the Pu-SN circuit, and this has been confirmed by multiple studies (Xicoy et al., 2020; Borra et al., 2021; Chang et al., 2021). However, as the disease exacerbates, cognitive functions might also be influenced, encompassing memory loss (Roheger et al., 2018). The sequential working memory might be affected with the connections of the Pu and STh, which provides a new neural circuit explanation framework for understanding cognitive impairments related to Parkinson’s disease (Ye et al., 2021).

The audio-motor control is relevant to the circuit of the VTA and Pu in bats (Schuller et al., 1997). Functional magnetic resonance imaging shows that abnormal functional connections between Broca’s and Wernicke’s areas and the Pu are associated with auditory hallucinations (Salisbury et al., 2021). Consistently, the caudal CP (CaT∼ and PuC∼) has strong and preferential connections with auditory thalamus (e.g., PIL and MGM) and cortex (A1 and A2) (Jiang and Kim, 2018; Cai et al., 2024; the present study). In addition, Pu appears to play roles in phonological processing, reading comprehension and speech articulation. The connections between the Pu and thalamus may be related with reading and organizing speech (Seghier and Price, 2010). For example, when the participants read meaningless syllables (Bohland and Guenther, 2006) or word stems (Rosen et al., 2000) aloud, the thalamus and Pu are more active than when they read silently.

## 5 Summary

This study has uncovered the existence of both CaT∼ and PuC∼ in the caudal striatum (“tail of the striatum”) of the rodents. This study has also systematically revealed the whole-brain afferent and efferent projections of the CaT∼ and PuC∼. The caudal striatum mainly receives its input from the SNC, SNL, VTA, PF, MG, PIL, VPM, and various sensorimotor and association cortical regions, and its efferent projections mainly target the GPe, GPi, SN, STh, and possibly VPM. Based on its brain-wide connections, the caudal striatum could play important roles in the integration of multimodal information, judgment of object value, habitual behavior, addiction and psychiatric disorders.

## Data Availability

The datasets presented in this study can be found in online repositories. The names of the repository/repositories and accession number(s) can be found in the article/Supplementary material.

## Footnotes

1
www.brain-map.org

2
https://gene-atlas.brainminds.jp/

3
http://connectivity.brain-map.org

## Supplementary material

Supplementary Figure 1 Expression of the genes *Otof* **(A)** and *Wfs1* **(B)** in the intermediate CP. At this level, these two genes are weakly and sparsely expressed in the PuI∼. In contrast, in the caudal CP, these two genes are strongly and densely expressed in the PuC∼ (see Supplementary Figure 2). Dashed lines outline the regional boundaries. Approximate bregma coordinates are indicated at the bottom right corner of each panel. Case IDs: 73788043 for *Otof* and 74881161 for *Wfs1*. Bar: 420μm in panel **(A)** for panels (**A** and **B**).

Supplementary Figure 2 Expression of the genes *Otof* and *Wfs1* in the caudal CP. **(A1–F1)** *Otof* expression in sequential coronal sections of the caudal CP. Slightly weaker *Otof* expression is seen in the CaT∼ than in the PuC∼. **(A2–F2)** *Wfs1* expression in sequential coronal sections of the caudal CP, matched with the *Otof* sections in **(A1–F1)**. Like *Otof*, slightly weaker *Wfs1* expression is seen in the CaT∼ than in the PuC∼. However, these two genes are differentially expressed in the central amygdaloid nucleus (Ce). Weak *Otof* and strong *Wfs1* expression are observed in the Ce, respectively. In addition, weaker *Otof* and stronger *Wfs1* expressions are found in the medioventral part (indicated by *) of the CaT∼ and PuC∼ as well as in the PuCv∼. Approximate bregma coordinates are indicated at the bottom right corner of each panel. Case IDs: 73788043 for *Otof* and 74881161 for *Wfs1*. Bar: 420μm in panel **(A1)** for all panels.

Supplementary Figure 3 Afferent connections of the caudal PuI∼ revealed with FG. **(A)** An FG injection (#) in the PuI∼ at bregma –0.70 mm. **(B–J)** Strongly labeled neurons are found in the SNC **(B,C)**, SNL **(B,C)**, VTA **(C)**, PIL **(B)**, S1BF **(D)**, S2 **(D)**, PF **(E)**, VPM **(E,F)**, STh **(F)**, MGM **(G)**, Ect- PRh-TeA **(H)**, M1 **(I)**, M2 **(I)**, AI **(I)** and FrA **(J)**. Note that layer 5 (L5) of the cortical areas contains most of the labeled neurons. The straight lines in panels **(D,H–J)** indicate approximate borders of the cortical areas. Approximate bregma coordinates are indicated at the top right corner of each panel. Bars: 500μm in panel **(A)** for all panels except panel F; 500μm in panel **(F)**.

Supplementary Figure 4 Determination of the extent of an FG injection site and labeled neurons. **(A–C)** Rostral-caudal extent of an FG injection (# in **A–C**) restricted in the rostral PuC∼. This case is the same one shown in Figure 5. The section shown in Figure 5A is located between panels **A** and **B** of this figure. In all these sections, the FG deposits are restricted in the PuC∼. Approximate bregma coordinates are indicated at the top right corner of panels **(A–C)**. **(D)** A higher magnification view of the FG labeled neurons in the M2 region shown in Figure 6D at Lower magnification. Two FG labeled neurons with typical neuronal morphology are indicated by arrows. Bars: 500μm in panel **(A)** for panels **(A–C)**; 100μm in panel **(D)**.

Supplementary Figure 5 Afferent connections of the PuC∼ revealed with FG. **(A–F)** Following an FG injection into the PuC∼ (# in **A**), labeled neurons are seen in the SNC **(B)**, VTA **(B)**, SNL **(B,D)**, MGM **(B,D)**, VPM **(C)**, MGV **(E,F)**. **(G-L)** Similar results are confirmed in another case with the FG injection in the PuC∼ (# in G). Note that, in these two cases, the tracer injections are not involved in the CaT∼. Approximate bregma coordinates are indicated at the top right corner of each panel. Bars: 200μm in **(A)** for panels **(A–F)**; 200μm in **(G)** for panels **(G–L)**.

Supplementary Figure 6 Projections from the PF to the CaT∼ (anterograde tracing). **(A,B)** Following an anterograde tracer injection into the laterodorsal PF (# in Inset in **A**) of a *Rbp4-Cre* mouse (ID: 300642574), labeled axon terminals are found mostly in the CaT∼ **(A,B)**. **(C,D)** Following the anterograde tracer injection into the lateral PF (# in Inset in **C**) of an *Efr3a-Cre* mouse (ID: 299732738), labeled axon terminals are also mostly observed in CaT∼ (**C,D**). Dashed lines indicate the borders between PuC∼ and GPe. Approximate bregma coordinates are indicated at the top right corner of each panel. Bars: 400μm in panel **(A)** for panels **(A–D)**; 560μm in the inset of panel **(A)**, also applies to the inset in panel **(C)**.

Supplementary Figure 7 Projections from the VPM to PuC∼ (anterograde tracing). **(A,B)** One tracer injection in the VPM (# in **A**) of a ppp1r17-Cre mouse (ID:268399868) produces strong axon terminal labeling in the S1 (mainly in layer 4) and PuC∼ **(B)** but not in the CaT∼, PuR∼ and PuI∼ (data not shown). In addition, very strongly labeled fiber bundles (fb) are observed through the PuC∼ to reach the S1. Approximate bregma coordinates are indicated at the top right corner of panels (**A** and **B**). **(C)** A high magnification view of the labeled axon terminals in the PuC∼. Note that the labeled axon terminals bear many varicosities (arrows). The asterisks in panels **(B)** and **(C)** indicate the same locations. In an additional wild-type case, the tracer injection in the VPM leads to similar results with no labeled axon terminals in VPM (data not shown). Bars: 280μm in **(A)**; 280μm in **(B)**; 50μm in **(C)**.

Supplementary Figure 8 Determination of the extent of an BDA injection site and labeled axon terminals. **(A–C)** Rostral-caudal extent of a BDA injection (# in **A–C)** restricted in the PuI∼. This case is the same one shown in Figure 11A. The section shown in Figure 11A is located between panels **B** and **C** of this figure. In all these sections, the BDA deposits are restricted in the PuI∼. Approximate bregma coordinates are indicated at the top right corner of panels **(A–C)**. **(D)** A higher magnification view of BDA labeled terminals in the VPM region shown in Figure 11H at lower magnification. **(E)** A higher magnification view of BDA labeled terminals in the SNL region shown in Figure 12G at lower magnification. **(F)** A higher magnification view of the virus labeled terminals in the SNL, resulted from the injection in CaT∼ (see inset in **F**) of a *Plxnd1-Cre* case (ID: 293366035). This injection also results in terminal labeling in GPi (see panel **G**). **(G)** Labeled axon terminals in the GPi show clearly visible varicosities (arrows) at high magnification. All these results confirm the terminal labeling from BDA tracing experiments. Bars: 500μm in panel **(A)** for panels **(A–C)**; 200μm in panel **(D)**; 100μm in panel **(E)**; 100μm in panel **(F)**; 560μm in the inset of panel **(F)**; 50μm in panel **(G)**.

Supplementary Figure 9 Projections from the GPe to thalamic nuclei (anterograde tracing). **(A–C)** One tracer injection in the dorsal GPe (# in **A**) of a *Pvalb-Cre* mouse (ID:511942270) leads to labeled axon terminals in Rt **(A)**, PF **(B)**, STh **(B)** and SNR **(C)** but not in VPM **(B)**. **(D–F)** Another tracer injection in the medial GPe (# in **E**) of a wild-type mouse (ID: 158373958) also leads to labeled axon terminals in Rt **(D)**, PF **(E)**, STh **(E)** and SNR **(F)** but not in VPM **(E)**. These results suggest that GPe does not project to VPM. Dashed lines outline the boundaries of the GPe and VPM. Approximate bregma coordinates are indicated at the bottom right corner of each panel. Bars: 280μm in panel **(A)** for all panels.

Supplementary Figure 10 Efferent projections of the caudal CP (anterograde tracing). **(A–B)** One tracer injection in the dorsal PuC∼ (# in **A**) of a *Gad2*-Cre mouse (ID: 157911832) produces labeled terminals in the ventrolateral SNR **(B)**, dorsal GPe and GPi **(C)**. **(D–F)** Another tracer injection in the CaT∼ (# in **D**) of a *Efr3a*-Cre mouse (ID: 301180385) leads to strongly and weakly labeled terminals in the ventrolateral SNR **(E)** and dorsal GPe **(F)**, respectively. GPi does not contain labeled terminals. Approximate bregma coordinates are indicated at the top right corner of each panel. Bars: 560μm in panel **(A)** for panels **(A,D)**; 400μm in panel **(B)** for panels **(B,C,E,F)**.

Supplementary Table 1 Semi-quantification of the relative density of the labeled neurons in the input source regions following FG injections into three rostral-caudal levels of the CP. −,+,++, and +++ indicate no, weak, medium and high density of labeled neurons, respectively.
