# Supplementary figures and images for: Localization and connections of the tail of caudate and caudal putamen in mouse brain
Source: Front Neural Circuits. 2025 Aug 4;19:1611199. doi: 10.3389/fncir.2025.1611199 (PMC12358408; doi:10.3389/fncir.2025.1611199)

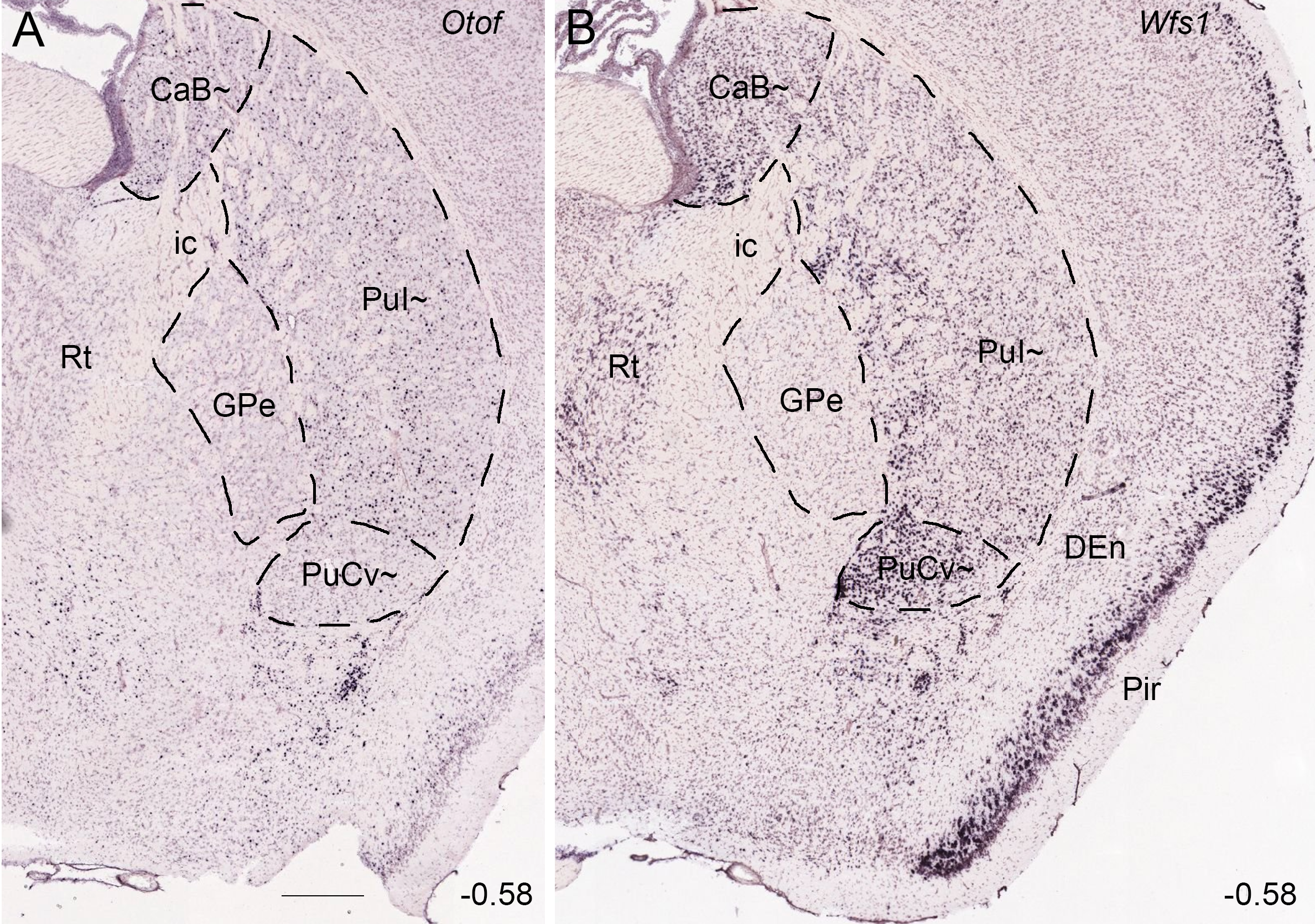

Supplement: Supplementary Figure 1 — Expression of the genes Otof (A) and Wfs1 (B) in the intermediate CP. At this level, these two genes are weakly and sparsely expressed in the PuI∼. In contrast, in the caudal CP, these two genes are strongly and densely expressed in the PuC∼ (see Supplementary Figure 2). Dashed lines outline the regional boundaries. Approximate bregma coordinates are indicated at the bottom right corner of each panel. Case IDs: 73788043 for Otof and 74881161 for Wfs1. Bar: 420μm in panel (A) for panels (A and B). [file Image_1.tif]

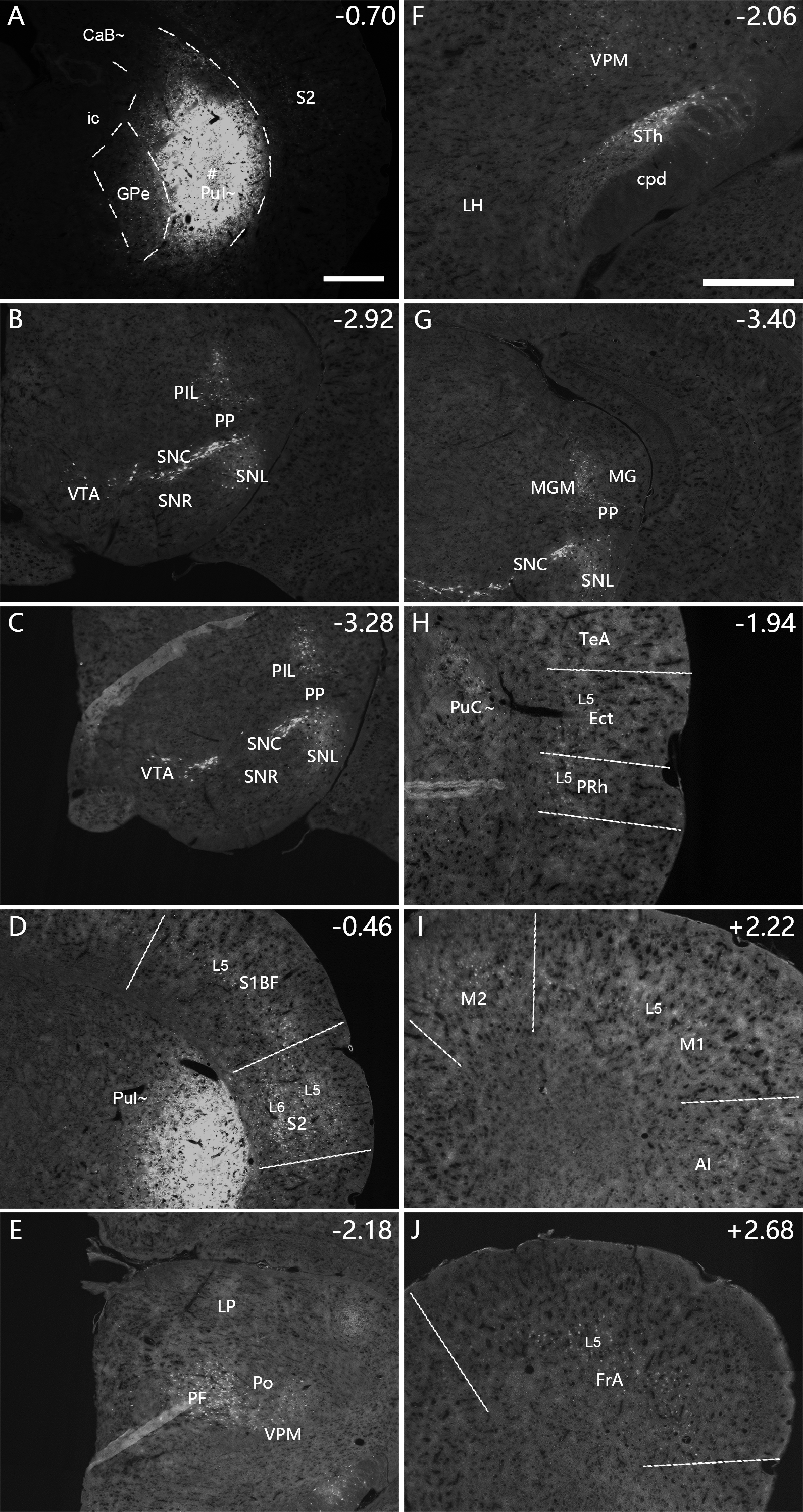

Supplement: Supplementary Figure 3 — Afferent connections of the caudal PuI∼ revealed with FG. (A) An FG injection (#) in the PuI∼ at bregma –0.70 mm. (B–J) Strongly labeled neurons are found in the SNC (B,C), SNL (B,C), VTA (C), PIL (B), S1BF (D), S2 (D), PF (E), VPM (E,F), STh (F), MGM (G), Ect- PRh-TeA (H), M1 (I), M2 (I), AI (I) and FrA (J). Note that layer 5 (L5) of the cortical areas contains most of the labeled neurons. The straight lines in panels (D,H–J) indicate approximate borders of the cortical areas. Approximate bregma coordinates are indicated at the top right corner of each panel. Bars: 500μm in panel (A) for all panels except panel F; 500μm in panel (F). [file Image_3.tif]

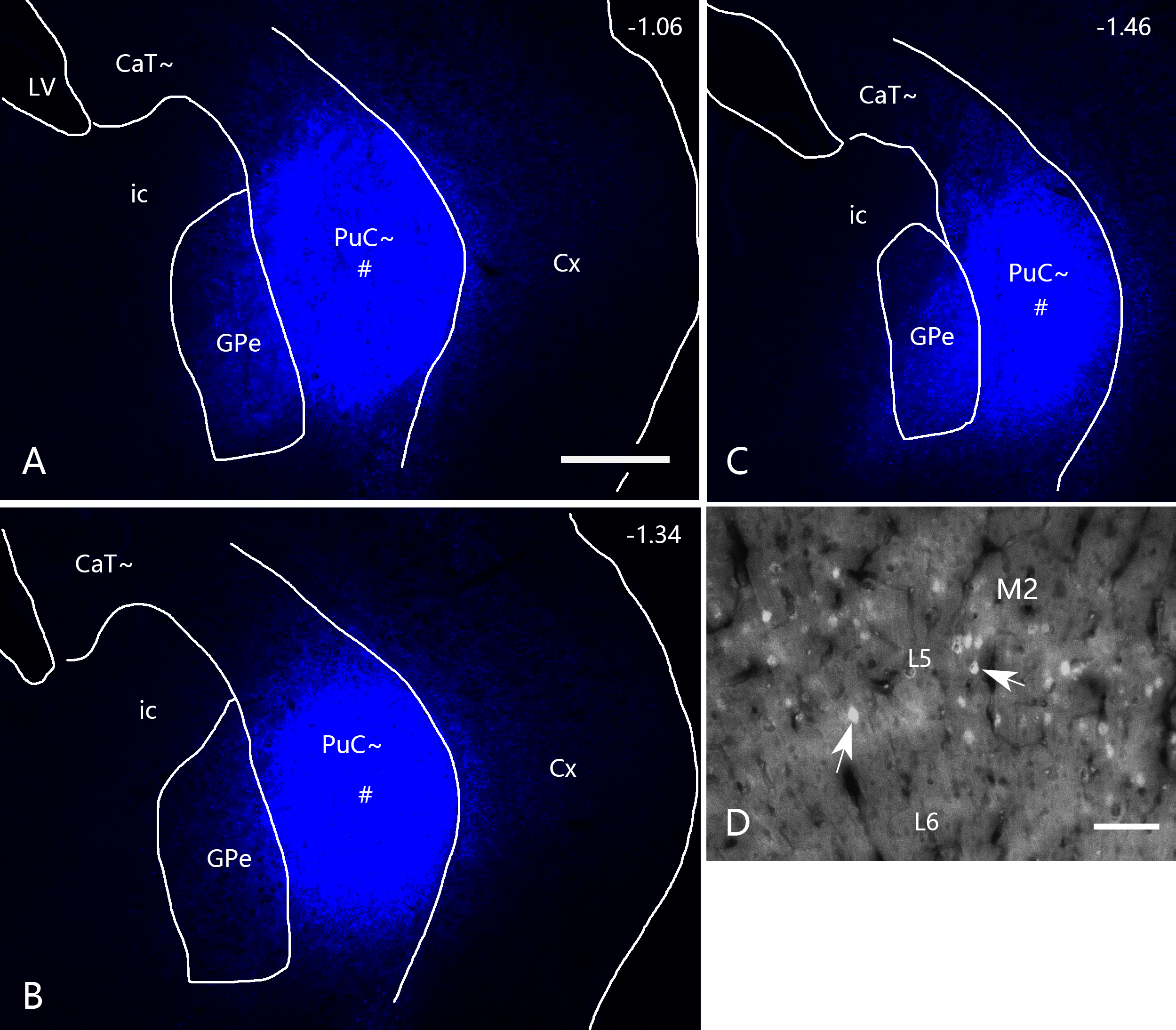

Supplement: Supplementary Figure 4 — Determination of the extent of an FG injection site and labeled neurons. (A–C) Rostral-caudal extent of an FG injection (# in A–C) restricted in the rostral PuC∼. This case is the same one shown in Figure 5. The section shown in Figure 5A is located between panels A and B of this figure. In all these sections, the FG deposits are restricted in the PuC∼. Approximate bregma coordinates are indicated at the top right corner of panels (A–C). (D) A higher magnification view of the FG labeled neurons in the M2 region shown in Figure 6D at Lower magnification. Two FG labeled neurons with typical neuronal morphology are indicated by arrows. Bars: 500μm in panel (A) for panels (A–C); 100μm in panel (D). [file Image_4.tif]

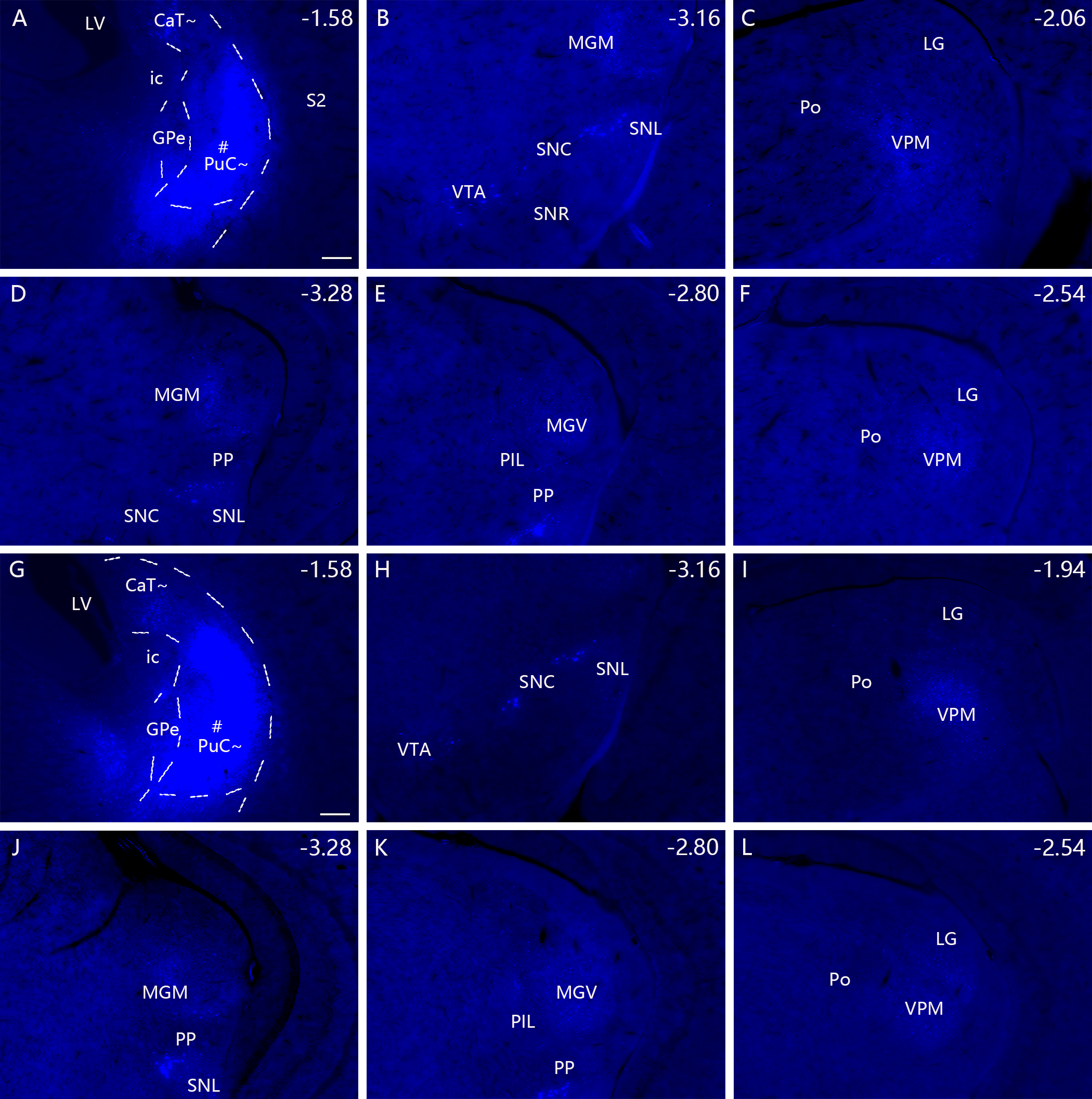

Supplement: Supplementary Figure 5 — Afferent connections of the PuC∼ revealed with FG. (A–F) Following an FG injection into the PuC∼ (# in A), labeled neurons are seen in the SNC (B), VTA (B), SNL (B,D), MGM (B,D), VPM (C), MGV (E,F). (G-L) Similar results are confirmed in another case with the FG injection in the PuC∼ (# in G). Note that, in these two cases, the tracer injections are not involved in the CaT∼. Approximate bregma coordinates are indicated at the top right corner of each panel. Bars: 200μm in (A) for panels (A–F); 200μm in (G) for panels (G–L). [file Image_5.tif]

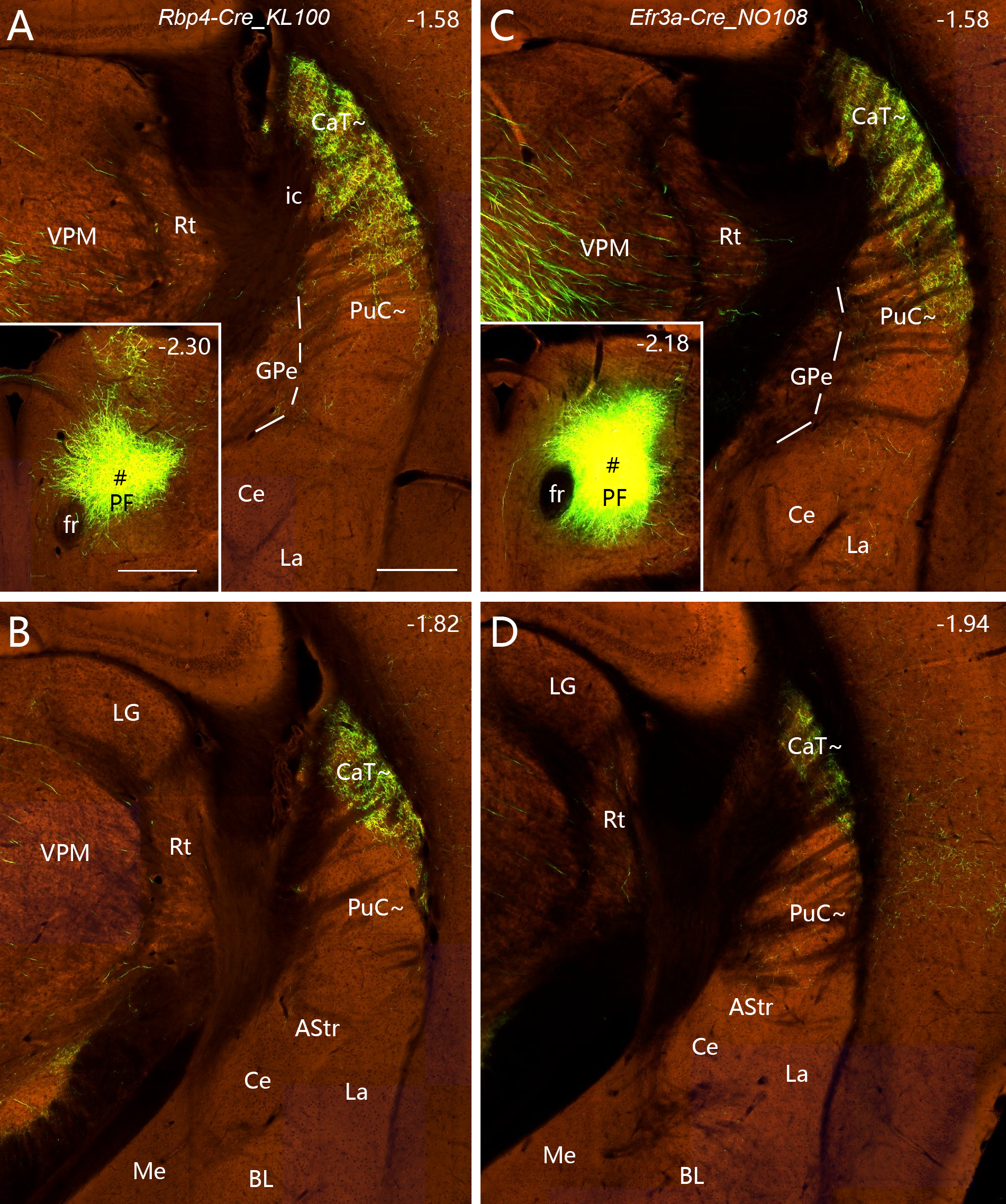

Supplement: Supplementary Figure 6 — Projections from the PF to the CaT∼ (anterograde tracing). (A,B) Following an anterograde tracer injection into the laterodorsal PF (# in Inset in A) of a Rbp4-Cre mouse (ID: 300642574), labeled axon terminals are found mostly in the CaT∼ (A,B). (C,D) Following the anterograde tracer injection into the lateral PF (# in Inset in C) of an Efr3a-Cre mouse (ID: 299732738), labeled axon terminals are also mostly observed in CaT∼ (C,D). Dashed lines indicate the borders between PuC∼ and GPe. Approximate bregma coordinates are indicated at the top right corner of each panel. Bars: 400μm in panel (A) for panels (A–D); 560μm in the inset of panel (A), also applies to the inset in panel (C). [file Image_6.tif]

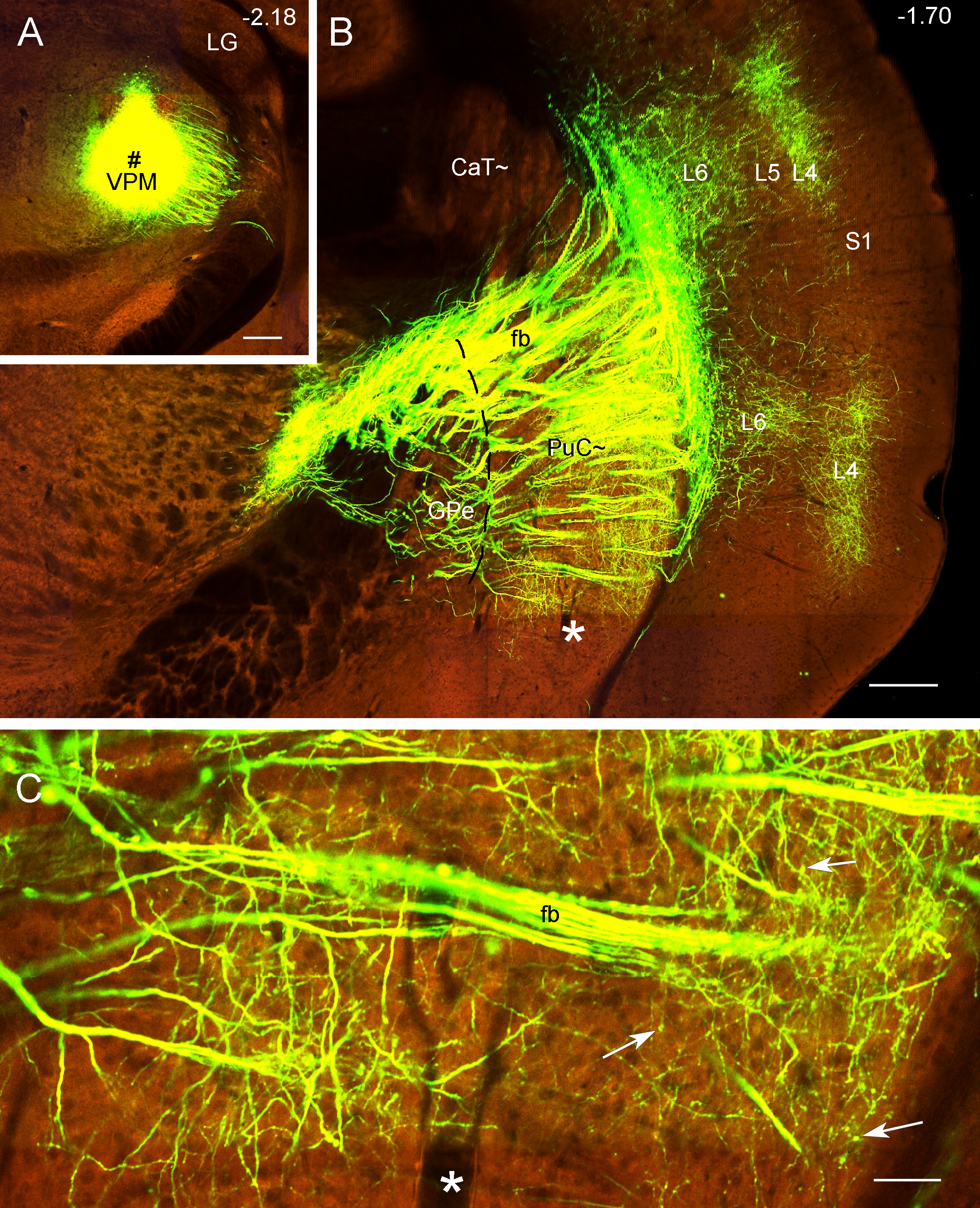

Supplement: Supplementary Figure 7 — Projections from the VPM to PuC∼ (anterograde tracing). (A,B) One tracer injection in the VPM (# in A) of a ppp1r17-Cre mouse (ID:268399868) produces strong axon terminal labeling in the S1 (mainly in layer 4) and PuC∼ (B) but not in the CaT∼, PuR∼ and PuI∼ (data not shown). In addition, very strongly labeled fiber bundles (fb) are observed through the PuC∼ to reach the S1. Approximate bregma coordinates are indicated at the top right corner of panels (A and B). (C) A high magnification view of the labeled axon terminals in the PuC∼. Note that the labeled axon terminals bear many varicosities (arrows). The asterisks in panels (B) and (C) indicate the same locations. In an additional wild-type case, the tracer injection in the VPM leads to similar results with no labeled axon terminals in VPM (data not shown). Bars: 280μm in (A); 280μm in (B); 50μm in (C). [file Image_7.tif]

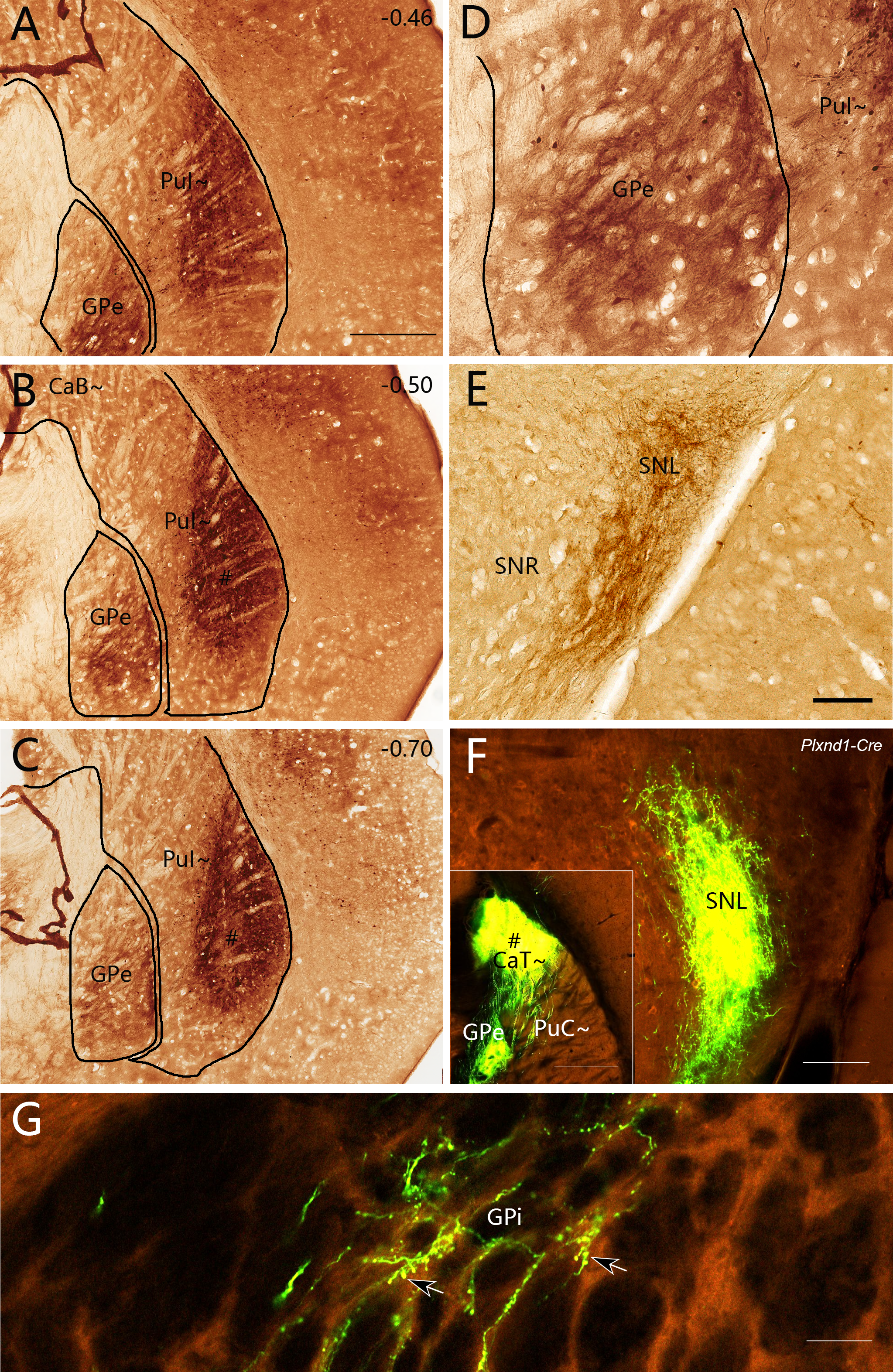

Supplement: Supplementary Figure 8 — Determination of the extent of an BDA injection site and labeled axon terminals. (A–C) Rostral-caudal extent of a BDA injection (# in A–C) restricted in the PuI∼. This case is the same one shown in Figure 11A. The section shown in Figure 11A is located between panels B and C of this figure. In all these sections, the BDA deposits are restricted in the PuI∼. Approximate bregma coordinates are indicated at the top right corner of panels (A–C). (D) A higher magnification view of BDA labeled terminals in the VPM region shown in Figure 11H at lower magnification. (E) A higher magnification view of BDA labeled terminals in the SNL region shown in Figure 12G at lower magnification. (F) A higher magnification view of the virus labeled terminals in the SNL, resulted from the injection in CaT∼ (see inset in F) of a Plxnd1-Cre case (ID: 293366035). This injection also results in terminal labeling in GPi (see panel G). (G) Labeled axon terminals in the GPi show clearly visible varicosities (arrows) at high magnification. All these results confirm the terminal labeling from BDA tracing experiments. Bars: 500μm in panel (A) for panels (A–C); 200μm in panel (D); 100μm in panel (E); 100μm in panel (F); 560μm in the inset of panel (F); 50μm in panel (G). [file Image_8.tif]

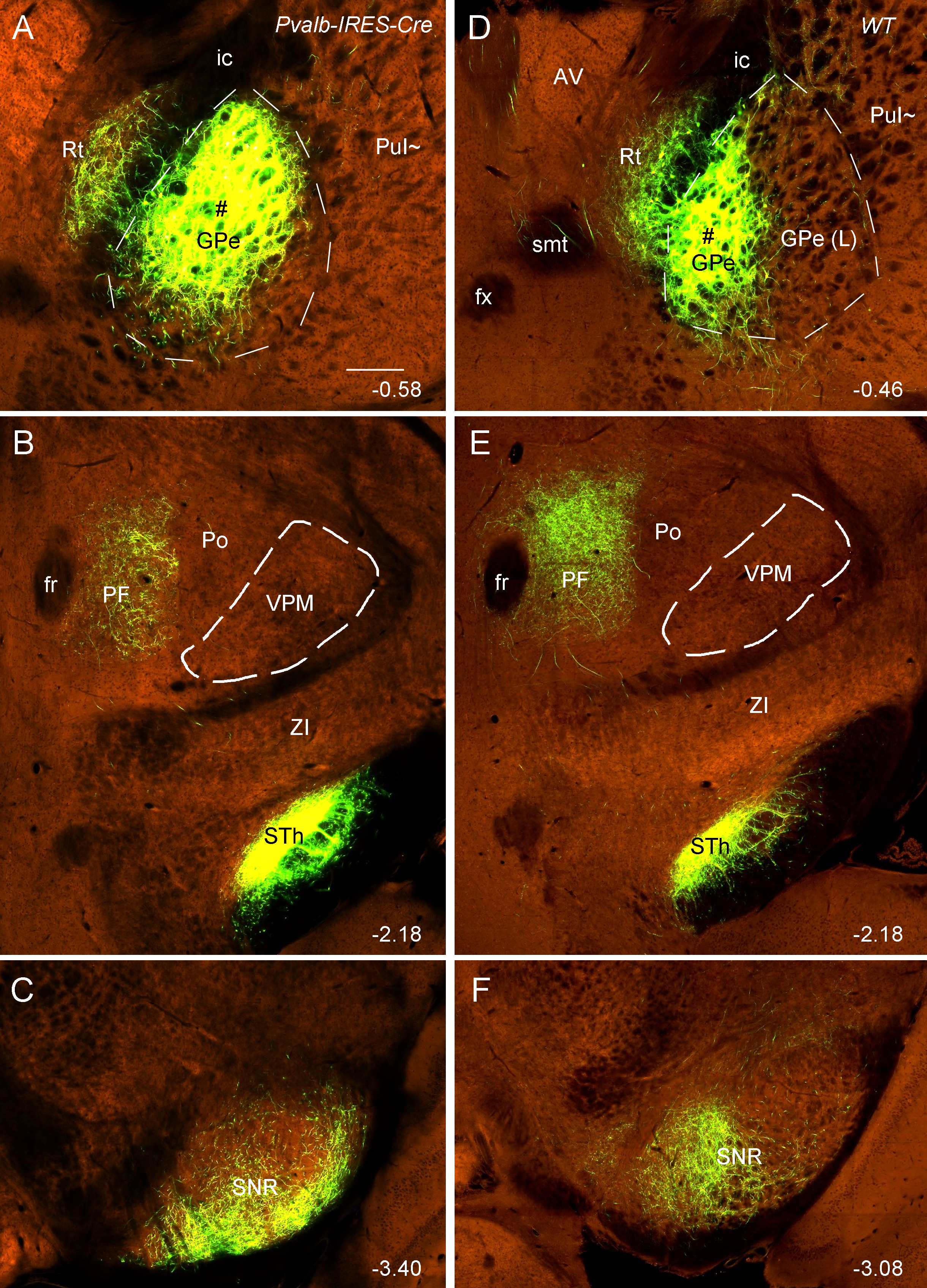

Supplement: Supplementary Figure 9 — Projections from the GPe to thalamic nuclei (anterograde tracing). (A–C) One tracer injection in the dorsal GPe (# in A) of a Pvalb-Cre mouse (ID:511942270) leads to labeled axon terminals in Rt (A), PF (B), STh (B) and SNR (C) but not in VPM (B). (D–F) Another tracer injection in the medial GPe (# in E) of a wild-type mouse (ID: 158373958) also leads to labeled axon terminals in Rt (D), PF (E), STh (E) and SNR (F) but not in VPM (E). These results suggest that GPe does not project to VPM. Dashed lines outline the boundaries of the GPe and VPM. Approximate bregma coordinates are indicated at the bottom right corner of each panel. Bars: 280μm in panel (A) for all panels. [file Image_9.tif]

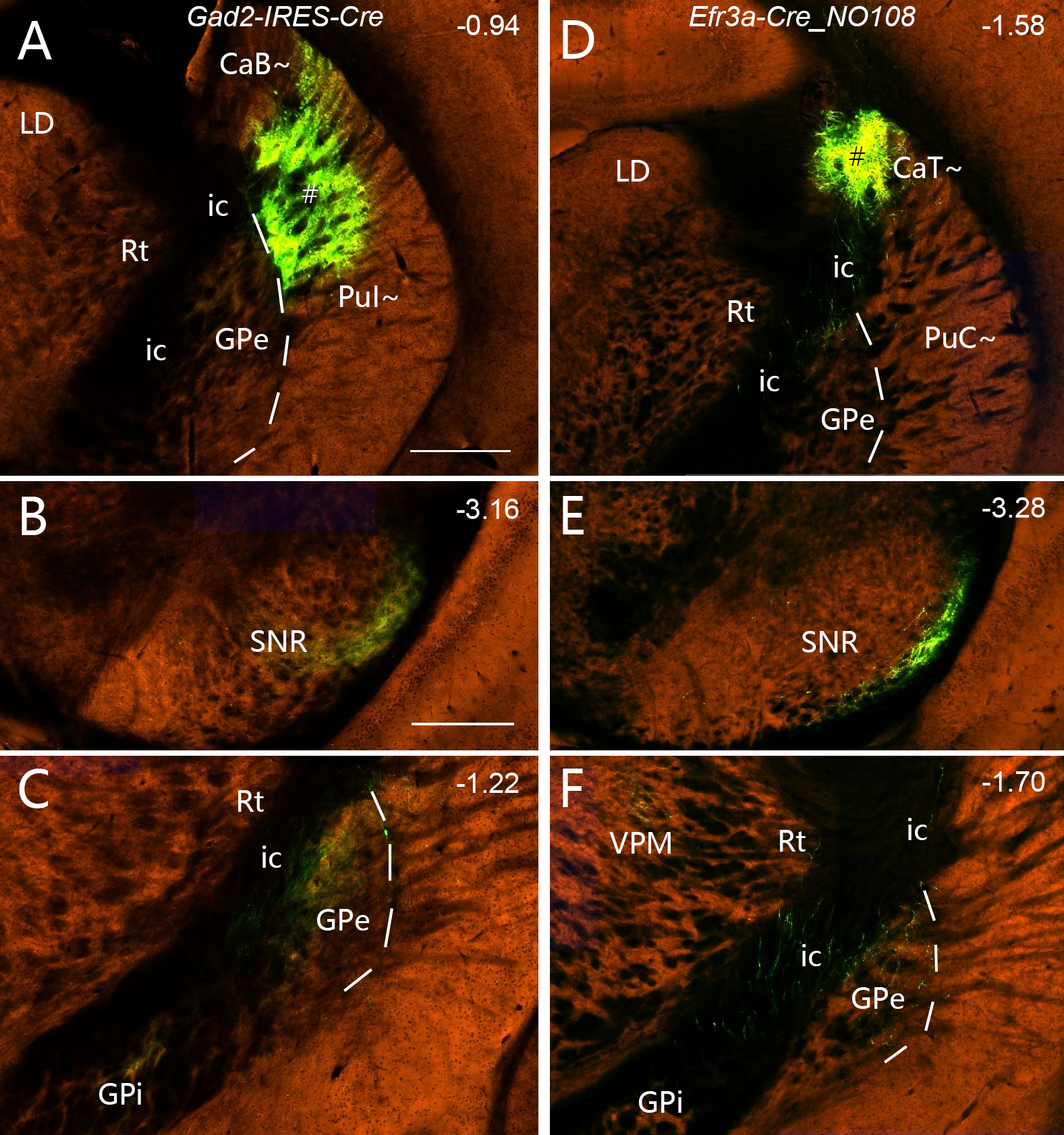

Supplement: Supplementary Figure 10 — Efferent projections of the caudal CP (anterograde tracing). (A–B) One tracer injection in the dorsal PuC∼ (# in A) of a Gad2-Cre mouse (ID: 157911832) produces labeled terminals in the ventrolateral SNR (B), dorsal GPe and GPi (C). (D–F) Another tracer injection in the CaT∼ (# in D) of a Efr3a-Cre mouse (ID: 301180385) leads to strongly and weakly labeled terminals in the ventrolateral SNR (E) and dorsal GPe (F), respectively. GPi does not contain labeled terminals. Approximate bregma coordinates are indicated at the top right corner of each panel. Bars: 560μm in panel (A) for panels (A,D); 400μm in panel (B) for panels (B,C,E,F). [file Image_10.tif]
