# Supplementary material for: Localization and connections of the tail of caudate and caudal putamen in mouse brain
Source: Front Neural Circuits. 2025 Aug 4;19:1611199. doi: 10.3389/fncir.2025.1611199 (PMC12358408; doi:10.3389/fncir.2025.1611199)
Supplement: Supplementary Figure 2 — Expression of the genes Otof and Wfs1 in the caudal CP. (A1–F1) Otof expression in sequential coronal sections of the caudal CP. Slightly weaker Otof expression is seen in the CaT∼ than in the PuC∼. (A2–F2) Wfs1 expression in sequential coronal sections of the caudal CP, matched with the Otof sections in (A1–F1). Like Otof, slightly weaker Wfs1 expression is seen in the CaT∼ than in the PuC∼. However, these two genes are differentially expressed in the central amygdaloid nucleus (Ce). Weak Otof and strong Wfs1 expression are observed in the Ce, respectively. In addition, weaker Otof and stronger Wfs1 expressions are found in the medioventral part (indicated by *) of the CaT∼ and PuC∼ as well as in the PuCv∼. Approximate bregma coordinates are indicated at the bottom right corner of each panel. Case IDs: 73788043 for Otof and 74881161 for Wfs1. Bar: 420μm in panel (A1) for all panels. [file Image_2.pdf]

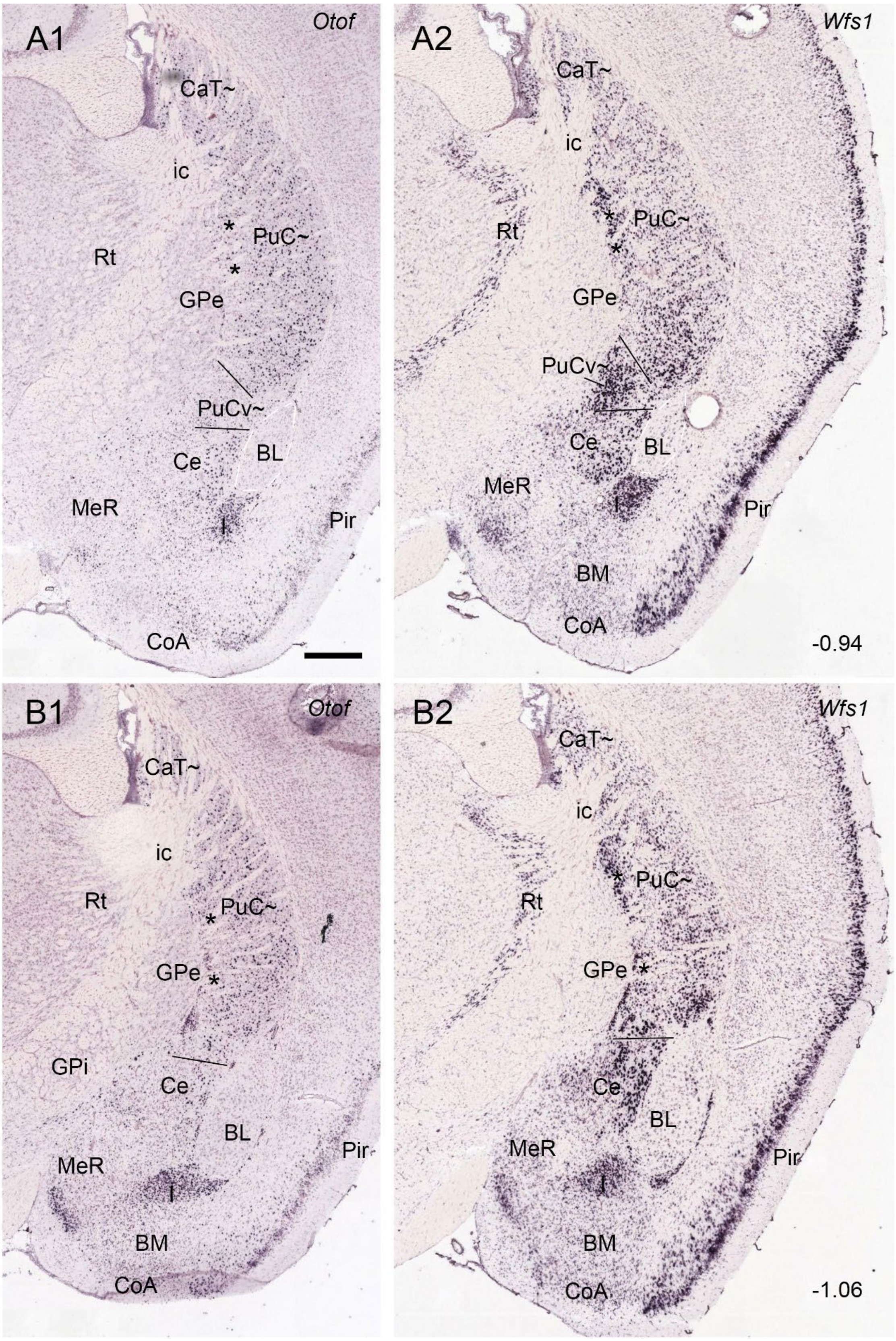
A1
Otof
CaT~
ic
Rt
PuC~
GPe
PuCv~
Ce
BL
MeR
I
Pir
CoA
A2
Wfs1
CaT~
ic
Rt
PuC~
GPe
PuCv~
Ce
BL
MeR
I
Pir
BM
CoA
-0.94
B1
Otof
CaT~
ic
Rt
PuC~
GPe
GPi
Ce
BL
MeR
I
Pir
BM
CoA
B2
Wfs1
CaT~
ic
Rt
PuC~
GPe
Ce
BL
MeR
I
Pir
BM
CoA
-1.06

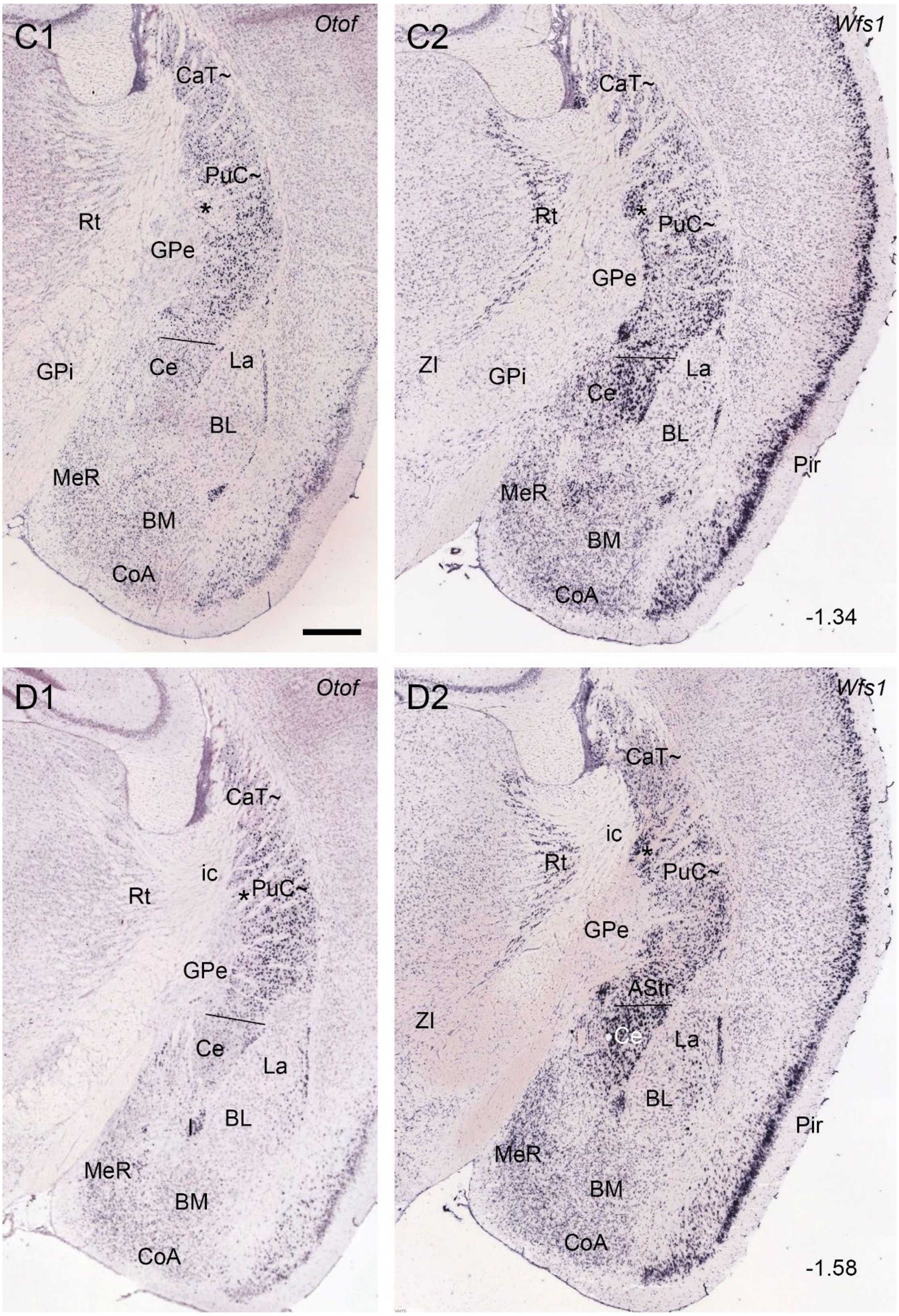
C1
Otof
CaT~
PuC~
*
Rt
GPe
GPi
Ce
La
BL
MeR
BM
CoA
C2
Wfs1
CaT~
Rt
*
PuC~
GPe
ZI
GPi
La
Ce
BL
Pir
MeR
BM
CoA
-1.34
D1
Otof
CaT~
ic
Rt
*PuC~
GPe
Ce
La
BL
MeR
BM
CoA
D2
Wfs1
CaT~
ic
Rt
*
PuC~
GPe
AStr
ZI
Ce
La
BL
Pir
MeR
BM
CoA
-1.58

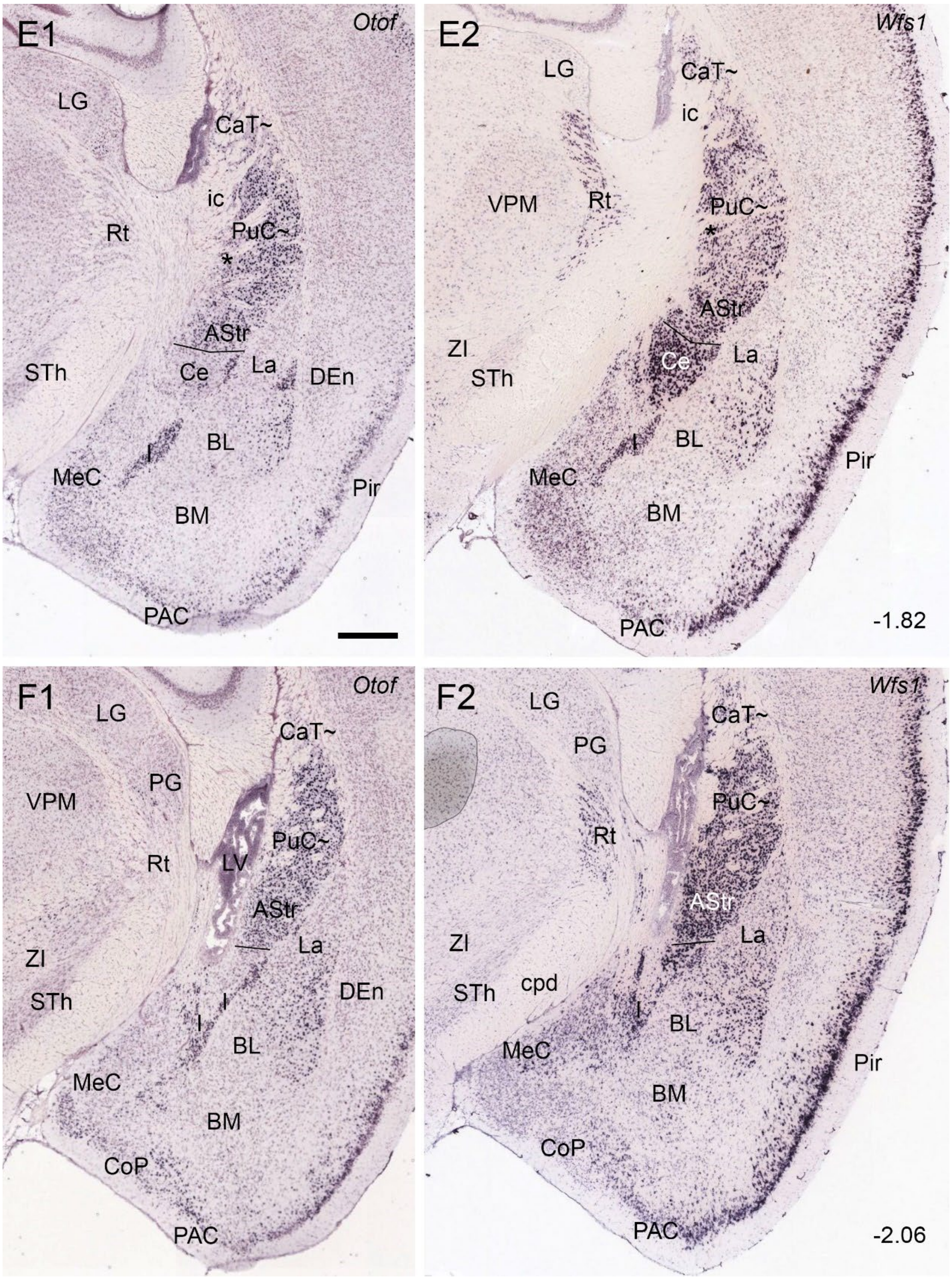

Suppl. Figure 2

Expression of the genes *Otof* and *Wfs1* in the caudal CP. (A1-F1) *Otof* expression in sequential coronal sections of the caudal CP. Slightly weaker *Otof* expression is seen in the CaT~ than in the PuC~. (A2-F2) *Wfs1* expression in sequential coronal sections of the caudal CP, matched with the *Otof* sections in (A1-F1). Like *Otof*, slightly weaker *Wfs1* expression is seen in the CaT~ than in the PuC~. However, these two genes are differentially expressed in the central amygdaloid nucleus (Ce). Weak *Otof* and strong *Wfs1* expression are observed in the Ce, respectively. In addition, weaker *Otof* and stronger *Wfs1* expressions are found in the medioventral part (indicated by *) of

the CaT~ and PuC~ as well as in the PuCv~. Approximate bregma coordinates are indicated at the bottom right corner of each panel. **Case IDs: 73788043 for *Otof* and 74881161 for *Wfs1*.** Bar: 420 µm in panel (A1) for all panels.
