# Supplementary material for: Localization and connections of the tail of caudate and caudal putamen in mouse brain
Source: Front Neural Circuits. 2025 Aug 4;19:1611199. doi: 10.3389/fncir.2025.1611199 (PMC12358408; doi:10.3389/fncir.2025.1611199)
Supplement: Supplementary Table 1 — Semi-quantification of the relative density of the labeled neurons in the input source regions following FG injections into three rostral-caudal levels of the CP. −,+,++, and +++ indicate no, weak, medium and high density of labeled neurons, respectively. [file Table_1.docx]

| Input source regions | PuI~ injections (AP -0.58 or -0.70mm) | PuC~ injections (AP -1.22mm) | PuC~ injections (AP: -1.58 or -1.70mm) | PuC~ and CaT~ injections (AP -1.58 or -1.70mm) |
| --- | --- | --- | --- | --- |
| S1 | **++** | **+++** | **+** | **++** |
| S2 | **+++** | **+++** | **-** | **++** |
| M1 | **+++** | **++** | **-** | **-** |
| M2 | **++** | **++** | **+** | **++** |
| AI | **++** | **++** | **+** | **++** |
| FrA | **+++** | **++** | **-** | **-** |
| LEC | **-** | **+** | **-** | **++** |
| Ect | **++** | **+++** | **+** | **++** |
| TeA | **++** | **++** | **+** | **++** |
| PRh | **++** | **++** | **-** | **++** |
| V1 | **-** | **-** | **-** | **++** |
| V2M | **-** | **-** | **-** |  |
| V2L | **-** | **-** | **-** | **+++** |
| Au1 (A1) | **-** | **-** | **+** | **++** |
| AuV | **-** | **+** | **++** | **+++** |
| AuD | **-** | **+** | **++** | **+++** |
| PPC | **-** | **-** | **+** | **++** |
| A29 (RSG) | **-** | **-** | **-** | **++** |
| A30 (RSA) | **-** | **-** | **-** | **++** |
| A23 | **-** | **-** | **-** | **++** |
| PF | **++** | **+++** | **+** | **++** |
| VPM | **+** | **++** | **++** | **++** |
| CM | **++** | **++** | **-** | **-** |
| MGM | **++** | **+++** | **++** | **++** |
| MGV | **-** | **-** | **++** | **++** |
| PIL | **++** | **+++** | **+++** | **++** |
| STh | **++** | **+++** | **-** | **-** |
| SNC | **+++** | **+++** | **++** | **++** |
| SNL | **++** | **++** | **++** | **++** |
| VTA | **+++** | **+++** | **++** | **++** |
| BL | + | + | + | +++ |
